# Transcriptome Dynamics Underlying Chlamydospore Formation in *Trichoderma virens* GV29-8

**DOI:** 10.3389/fmicb.2021.654855

**Published:** 2021-06-08

**Authors:** Xinhong Peng, Beilei Wu, Shuaihu Zhang, Mei Li, Xiliang Jiang

**Affiliations:** Institute of Plant Protection, Chinese Academy of Agricultural Sciences, Beijing, China

**Keywords:** *Trichoderma virens*, chlamydospores, transcriptome, GO enrichment, KEGG enrichment, STC analysis, WGCNA analysis

## Abstract

*Trichoderma* spp. are widely used biocontrol agents which are antagonistic to a variety of plant pathogens. Chlamydospores are a type of propagules produced by many fungi that have thick walls and are highly resistant to adverse environmental conditions. Chlamydospore preparations of *Trichoderma* spp. can withstand various storage conditions, have a longer shelf life than conidial preparations and have better application potential. However, large-scale production of chlamydospores has proven difficult. To understand the molecular mechanisms governing chlamydospore formation (CF) in *Trichoderma* fungi, we performed a comprehensive analysis of transcriptome dynamics during CF across 8 different developmental time points, which were divided into 4 stages according to PCA analysis: the mycelium growth stage (S1), early and middle stage of CF (S2), flourishing stage of CF (S3), and late stage of CF and mycelia initial autolysis (S4). 2864, 3206, and 3630 DEGs were screened from S2 vs S1, S3 vs S2, and S4 vs S3, respectively. We then identified the pathways and genes that play important roles in each stage of CF by GO, KEGG, STC and WGCNA analysis. The results showed that DEGs in the S2 vs S1 were mainly enriched in organonitrogen compound metabolism, those in S3 vs S2 were mainly involved in secondary metabolite, cell cycle, and N-glycan biosynthesis, and DEGs in S4 vs S3 were mainly involved in lipid, glycogen, and chitin metabolic processes. We speculated that mycelial assimilation and absorption of exogenous nitrogen in the early growth stage (S1), resulted in subsequent nitrogen deficiency (S2). At the same time, secondary metabolites and active oxygen free radicals released during mycelial growth produced an adverse growth environment. The resulting nitrogen-deficient and toxin enriched medium may stimulate cell differentiation by initiating cell cycle regulation to induce morphological transformation of mycelia into chlamydospores. High expression of genes relating to glycogen, lipid, mannan, and chitin synthetic metabolic pathways during the flourishing (S3) and late stages (S4) of CF may be conducive to energy storage and cell wall construction in chlamydospores. For further verifying the functions of the amino sugar and nucleotide sugar metabolism (tre00520) pathway in the CF of *T. virens* GV29-8 strain, the chitin synthase gene (TRIVIDRAFT_90152), one key gene of the pathway, was deleted and resulted in the dysplasia of mycelia and an incapability to form normal chlamydospores, which illustrated the pathway affecting the CF of *T. virens* GV29-8 strain. Our results provide a new perspective for understanding the genetics of biochemical pathways involved in CF of *Trichoderma* spp.

## Introduction

Fungi in the genus *Trichoderma* are widely applied bio-control agents which are inhibitory to a variety of plant pathogens (Harman, 2006; Steindorff et al., 2014; Vos et al., 2015; Kim and Vujanovic, 2016; Deng et al., 2018). Currently, commercially available *Trichoderma* formulations primarily use conidiospore preparations, which can experience reductions in viability under nonoptimal storage conditions. Chlamydospores are a type of large, spherical, thick-walled spore produced by numerous fungi, including *Trichoderma* spp., under adverse environmental conditions (Hsu, 1973; Griffin, 1976; Ment et al., 2010; Agustí-Brisach and Armengol, 2012; Li et al., 2012; Navarathna et al., 2016; Sun et al., 2019; Yuan et al., 2019). Due to these properties, chlamydospore-based bio-control preparations could offer a longer shelf-life and higher bio-control capabilities against plant diseases than conidiospore-based preparations (Mishra et al., 2012; Zhang et al., 2017; Yuan et al., 2019). However, their application is limited by the difficulty of producing large numbers of chlamydospores. Research thus far has mainly focused on optimizing culture conditions to promote chlamydospore formation (CF) by *Trichoderma* (Zou et al., 2006; Mishra et al., 2012; Zhang et al., 2015; Li et al., 2016), but few molecular mechanisms underlying CF in *Trichoderma* have been explored (Yang et al., 2015; Yuan et al., 2019). Therefore, a comprehensive understanding of the molecular mechanisms regulating CF in *Trichoderma* could benefit the development of *Trichoderma* chlamydospore preparations.

CF involves multiple, complicated biological processes and pathways. High-resolution transcriptome sequencing technology has been utilized in single-celled eukaryotes, such as the yeast *Candida albicans*, providing insights into the molecular pathways and networks, along with their interactions, that are involved in various aspects of CF (Sonneborn et al., 1999; Alonso-Monge et al., 2003; Nobile et al., 2004; Eisman et al., 2006; Palige et al., 2013; Böttcher et al., 2016; Giosa et al., 2017). It was reported that the TOR, cAMP and MAPK signal pathways are very important for inducing CF in *C. albicans* (Alonso-Monge et al., 2003; Nobile et al., 2004; Staib and Morschhäuser, 2005; Böttcher et al., 2016). Other studies have shown that chitinase, chitin synthase, glucanase, and glycosyltransferase genes, as well as genes involved in amino sugar and nucleotide sugar metabolism, starch and sucrose metabolic pathways were speculated to be involved in CF in *T*. *harzianum* (Yang et al., 2015; Yuan et al., 2019). However, little other research has been performed to dissect the molecular mechanisms underlying CF in *Trichoderma* spp. To understand such mechanisms, we used RNA-seq to determine the transcriptomics of CF in *T. virens* GV29-8 at different developmental stages, hoping to reveal transcriptome-based dynamics of CF, and identify key determining factors of CF in *T*. *virens*. The transcript modules of co-expressed genes at different stages of CF and the series test of cluster (STC) analysis of differential time points during gene expression were performed to identify candidate genes and pathways that might determine CF. Furthermore, we created a knockout mutant of those genes to verify their functions in CF. This study provides insights into the molecular mechanisms underlying CF in *T*. *virens*.

## Materials and Methods

### Strain Culture and Sampling

The *T*. *virens* strain GV29-8 was used (Baek and Kenerley, 1998; Kubicek et al., 2011; Morán-Diez et al., 2015). It was purchased from the fungal genetics stock center (FGSC 10586, United States) and stored as conidial suspensions in 20% glycerol at -80 °C. The strain was inoculated onto a PDA plate, activated at 28°C for 3 days, then transferred to a fresh PDA plate and cultured at 28°C for 7 days. Conidia were harvested and adjusted to 1 × 10^7^ conidia mL^–1^ with sterile distilled water, 3 mL of which was added into 120 mL of chlamydospore inducing medium (glucose 1%, cornmeal 1%, yeast extract powder 0.5%, corn steep powder 1% and inductive agent 5%) in 500 mL Erlenmeyer flasks, and incubated at 28°C on a rotary shaker (180 rpm) in the dark for 3 days. Glucose was obtained from Beijing Chemical Works, yeast extract powder from Thermo Fisher Oxoid, cornmeal from Xinghua Yufeng Food Co., Ltd., and corn steep powder was supplied by Shandong Weiduofeng Biotechnology Co. Ltd. Inductive agent was explored by our lab.

Chlamydospore formation was observed using a microscope (Olympus BX53, Tokyo, Japan) every one to two hours, over the course of 3 days. Eight sampling time points were selected; samples were collected with 3 biological replicates at 20, 24, 26, 28, 32, 38, 45, and 56 h, represented by the labels TVS1, TVS2, TVS3, TVS4, TVS5, TVS6, TVS7, and TVS8, respectively. Samples were harvested by filtration and washed 3 times with sterile water. Residual water was removed using sterile filter paper. Samples were frozen in liquid nitrogen and used for transcriptome sequencing.

### Construction of cDNA Libraries and Transcriptome Sequencing

We commissioned Nuohe Zhiyuan Technology Co., Ltd., to conduct transcriptome sequencing using the Illumina NovaSeq 6000 platform. Total RNAs from each sample were extracted with Trizol reagent, using the manufacturer’s protocol. RNA concentration and quality were assessed using agarose gel electrophoresis, Nanodrop, Qubit 2.0 and the Agilent 2100 Bioanalyzer. After total RNA was extracted and quality tested, RNA-seq libraries were constructed using the NEBNext UltraTM RNA Library Prep Kit for Illumina (NEB). Briefly, after mRNA fragmentation, random primers were used for first-strand cDNA synthesis through M-MuLV reverse transcriptase system, before synthesis of second-strand cDNA, to obtain double-stranded cDNA. End-repair and addition of adenines to the 3′terminus of the double-stranded cDNA was performed, followed by ligation of sequencing adapters. Ligated products were purified and subjected to PCR amplification; PCR products were again purified with AMPure XP beads, to produce the final library preparation. The Agilent Bioanalyzer 2100 system was used for quality control of the libraries prior to loading on the machine for sequencing.

### Gene Annotation and Quantitative Analysis of Expression Levels

Clean reads were obtained by removing adapter sequences and filtering out low quality and poly-N containing reads (Chao et al., 2019). Next, clean reads were mapped onto the *T. virens* GV29-8 genome^1^. Gene expression levels were expressed as fragments per kilobase of transcript per million fragments mapped (FPKM), i.e., the number of matches for every kilobase of transcript per million fragments. Correlation among biological replicates was determined via the Pearson correlation coefficient, PCA, and phylogenetic analysis, which were performed using the R software package.

### Analysis of Differentially Expressed Genes

Differentially expressed genes (DEGs) between samples were identified using Deseq2 software, in which the fold-change was the ratio of the expression levels between two samples: i.e., log_2_^(*FC*)^ was log_2_^(*fold–change*)^ = log_2_^(*Sample A/Sample B*)^. Here, we set the *p*-value < 0.05 and | log_2_^(*FC*)^| > 0 as significant differences.

### GO Functional Classification and KEGG Pathway Enrichment Analysis of DEGs During CF in *T*. *virens* GV29-8

Gene ontology (GO) and Kyoto encyclopedia of genes and genomes (KEGG) pathway enrichment analyses for DEGs were performed using the Clusterprofiler software. The GO terms exhibiting a corrected (after adjusting with false discovery rate) *p*-value < 0.05 were considered significantly enriched. We used *T. reesei* as a reference for the KEGG pathway significant enrichment analysis with a p-value<0.05, because there was no information of *T. virens* in the KEGG database.

### STC and WGCNA Analysis During CF in *T. virens* GV29-8

Series test of cluster analysis was conducted based on Pearson correlations of gene expression profiles. To find trends in characteristics of gene expression and consolidate genes with the same characteristics into one trend, to produce the most representative gene group involved in CF. STC was implemented entirely in Java. Portions of the STC interface were implemented using the third-party library, the Java Piccolo toolkit from the University of Maryland (Bederson et al., 2004). STC also makes use of external Gene Ontology and gene annotation files. We specified a tab delimited gene expression data file as input to STC. Following the input phase, the STC clustering algorithm is executed and a new window will appear displaying the clustering results.

The weighted correlation network (WGCNA) analysis package in R (version 3.5.0) was used to construct a co-expression network for the filtered genes. After sample clustering, scale independence and mean connectivity analysis of modules with different power values was performed to determine the soft threshold of module analysis. The power value was set from 1 to 30, and values of scale independence and mean connectivity were generated according to these power values. The power value was determined when the scale independence value was 0.8. A hierarchical clustering dendrogram of the TOM matrix was constructed by the average distance with a minimum size threshold of 30 and the merge cut height of 0.25 to classify similar genes expression profiles into different gene modules. A cluster dendrogram among modules and an eigengene adjacency heatmap between modules were generated. Co-expression networks were visualized using the igraph package in R, which was also used to determine the betweenness of modules. Module-trait relationships were calculated according to the correlation between modules and traits, modules that were significantly correlated with individual traits (*P* < 0.05) were identified, and genes in significant modules were exported for further analysis.

### Validation of Transcriptome Data Using Reverse Transcription Quantitative PCR (RT-qPCR)

cDNA was synthesized from 1 μg of total RNA extracted using Trizol reagent, and 2 ul of cDNA was subsequently used as template for RT-qPCR. Gene-specific primers were designed using Primer 5 (v3.0) software (Supplementary Table 1). The product annotations are listed in Supplementary Table 1. The RT-qPCR reaction was performed on a 7500 Real-Time PCR System (Applied Biosystems, United States) using TB Green^®^ Premix ExTaq^TM^ (TaKaRa, China) according to the manufacturer’s instructions. The amplification conditions were 95°C for 30 s, followed by 40 cycles of 95°C for 5 s and 60°C for 34 s. Three biological replicates and at least 3 technical replicates were used for each sample. Normalization of transcript levels for each gene was done against transcript levels of the internal control gene glyceraldehyde-3-phosphate dehydrogenase (*GAPDH*), and fold changes were calculated using the 2^–ΔΔ*Ct*^ method (Livak and Schmittgen, 2001). RT-qPCR results were compared with the RNA-Seq data to detect the correlation of each gene expression.

### Analysis of Glycogen, Lipid, and Chitin Contents During CF in *T*. *virens* GV 29-8

Samples were collected across 8 time points (TVS1, TVS2, TVS3, TVS4, TVS5, TVS6, TVS7, and TVS8), using 3 biological replicates per sample. Samples were observed using a microscope (Olympus BX53, Tokyo, Japan) with bright-field optics, or appropriate filter sets for fluorescent visualization. A solution containing 60 mg mL^–1^ KI and 10 mg mL^–1^ I_2_ in distilled water was used for glycogen detection and observed in the bright-field optics (Seong et al., 2008). Nile Red solution (Sigma, N-3013) (0.01 mg mL^–1^ in acetone) was used for lipid detection (Son et al., 2012). A filter (excitation 543; emission 598) was applied for visualization of lipids. Chitin staining was performed by adding 2 μL of calcofluor white stock solution (10 mg mL^–1^, Sigma, 18909) to 20 μL of mycelia and chlamydospore samples placed on glass slides. which were then incubated for 15 min at 4°C, after which mycelia and chlamydospores were observed using a filter (excitation 355, emission 445) (Yu et al., 2008).

### Effect of Chitin Synthase Gene (TRIVIDRAFT_90152) Knockout on CF in *T. virens* GV29-8

To verify the contribution of the chitin synthase gene (*Chs*) (TRIVIDRAFT_90152) to CF in *T. virens* GV29-8, the *Chs* deletion mutant strain, *Chs*Δ, was generated using the split-PCR strategy (Catlett et al., 2003). The primers used are listed in Supplementary Table 26. The hygromycin resistance gene (*hyg*), which was amplified from the plasmid pKH-KO, conferred hygromycin resistance on the fungus. The protoplast transformation was used to generate the *Chs*Δ by transforming two fragments containing homologous region sequences of the *Chs* gene and partial hygromycin gene fragment into the protoplasts of wild-type *T. virens* GV29-8 (Aragona and Valente, 2015; Li et al., 2017). The genotypes of *Chs*Δ mutants were confirmed by amplifying internal fragment of *Chs* (no PCR product generated), and the *hyg* fragment (PCR product was 719 bp in size). Southern hybridization was performed using a DIG High prime DNA labeling and detection starter kit II (Roche, Germany) according to the manufacturer′s protocol. The *Chs* (407bp) gene fragment was amplified as the probe. The primers of the probe are listed in Supplementary Table 26. DNAs of wild-type *T. virens* GV29-8 and *Chs*Δ mutant strains were digested by BamHI/NotI.

The wild-type *T. virens* GV29-8 and mutant *Chs*Δ strain were each inoculated onto the center of PDA plates and cultured for 7 days at 28°C. Conidia of wild-type *T. virens* GV29-8 and *Chs*Δ strain were harvested and adjusted to 1 × 10^6^ conidia mL^–1^ with sterile distilled water, 3 mL of which was added into 120 mL of chlamydospore inducing medium and incubated at 28°C on a rotary shaker (180 rpm), and chlamydospore formation of each culture was observed by microscopy 4 days after inoculation. To determine the chitin content of chlamydospores and mycelia in wild-type *T. virens* GV29-8 and *Chs*Δ strain, calcofluor white staining was performed as previously described (Yu et al., 2008). Oil red O staining solution was used to determine lipid content (Shin et al., 2011).

### Statistical Analysis

Individual means and standard deviation of the mean were calculated using the data from independent samples in Microsoft Excel 2007 (Microsoft, United States). IBM SPSS statistics software (SPSS 17.0, United States) was used for correlation coefficient analysis. The correlation coefficient (*r*) between RNA-Seq and RT-qPCR was calculated using a two-tailed *p*-value with a confidence interval of 95%.

## Results

### Global Transcriptome Analysis of CF in *T*. *virens* GV29-8

**FIGURE 1:**
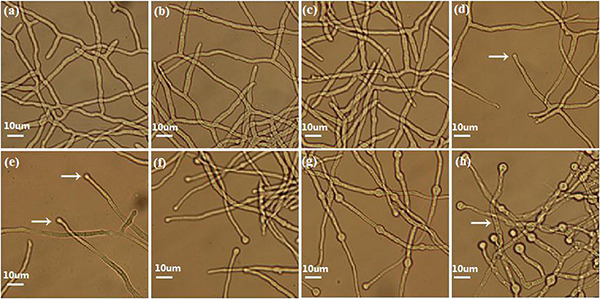
Phenotype of CF at different stages in *Trichoderma virens* GV29-8. No chlamydospores were produced in TVS1 (20 h), TVS2 (24 h) and TVS3 (26 h) as shown in pictures **(a)**, **(b)**, and **(c)**, respectively. **(d)** Chlamydospores production in mycelium tips began at TVS4 (28 h) (arrow). **(e)** Chlamydospores began to appear on the tips of a small number of mycelia in TVS5 (32 h) (arrow). **(f)** About half of the mycelium tips produced chlamydospores in TVS6 (38 h). **(g)** Almost all mycelium tips produced chlamydospores and a lot of internal chlamydospore production was also observed in TVS7 (45 h). **(h)** Mycelia began autolysis in TVS8 (56 h) (arrow). Scale bars = 10 μm.

Eight morphologically significant time point samples (TVS1, TVS2, TVS3, TVS4, TVS5, TVS6, TVS7, and TVS8) that represented significant events during CF were selected for transcriptome sequencing (Figure 1). The TVS1, TVS2 and TVS3 time point samples were collected during the mycelium growth stage. TVS4 and TVS5 samples were collected during the early stage of CF; chlamydospores were initially observed in TVS4 and became more obvious in TVS5. TVS6 and TVS7 samples were collected during the middle and flourishing stages of CF, respectively. About half of the mycelium tips produced chlamydospores in TVS6. All mycelium tips and parts of the mycelium interior produced chlamydospores in TVS7. Mycelia autolysis occurred during TVS8, at which point the mycelia had become vacuolated (Figure 1h).

We performed RNA-seq experiments using total RNA extracted from samples across the 8 selected time points. More than 688 million high quality reads (average 28 million reads from each sample) and 206.08 G of clean bases (average 8.59 G of data for each sample) were generated from different samples. Mapping to the *T*. *virens* GV29-8 genome (GenBank assembly accession: *GCA_000170995.2*), the alignment rate (total map) reached 79.34% ∼ 85.96%, among which sequences with unique alignment positions along the reference sequence reached over 79.07% (Table 1).

**TABLE 1:** Statistical analysis of sequencing results.

**Sample**	**Raw reads**	**clean reads**	**Clean bases**	**Q20(%)**	**Q30(%)**	**total_reads**	**total_map (%)**	**unique_map (%)**
TVS1_1	29417905	28921539	8.68G	97.73	93.68	57843078	49722058(85.96%)	49494155(85.57%)
TVS1_2	24816348	24317521	7.3G	97.03	92.2	48635042	41416437(85.16%)	41214532(84.74%)
TVS1_3	28808008	28215691	8.46G	97.13	92.41	56431382	47589649(84.33%)	47343663(83.9%)
TVS2_1	32787554	32288499	9.69G	97.17	92.48	64576998	54731573(84.75%)	54530206(84.44%)
TVS2_2	35585217	35013599	10.5G	97.19	92.56	70027198	58382659(83.37%)	58153313(83.04%)
TVS2_3	23229622	22870748	6.86G	96.7	91.45	45741496	38116549(83.33%)	37960918(82.99%)
TVS3_1	29495711	28581672	8.57G	97.3	92.83	57163344	46864148(81.98%)	46647373(81.6%)
TVS3_2	34168038	33519114	10.06G	97.24	92.65	67038228	55014001(82.06%)	54761803(81.69%)
TVS3_3	29082289	28641615	8.59G	97.74	93.72	57283230	47892671(83.61%)	47640072(83.17%)
TVS4_1	33371573	32170556	9.65G	96.91	91.99	64341112	52134998(81.03%)	51872009(80.62%)
TVS4_2	35623406	35011956	10.5G	97.12	92.4	70023912	57308188(81.84%)	57037980(81.46%)
TVS4_3	29058932	28280180	8.48G	96.72	91.53	56560360	46165285(81.62%)	45954745(81.25%)
TVS5_1	29231630	28686006	8.61G	97.54	93.29	57372012	46176229(80.49%)	45993516(80.17%)
TVS5_2	25794278	25394091	7.62G	97.01	92.14	50788182	40679800(80.1%)	40541740(79.83%)
TVS5_3	32493581	31550095	9.47G	97.13	92.46	63100190	50060893(79.34%)	49890603(79.07%)
TVS6_1	29930540	29346925	8.8G	97.21	92.58	58693850	47151572(80.33%)	46997999(80.07%)
TVS6_2	24223306	23792470	7.14G	97.15	92.43	47584940	38399674(80.7%)	38272844(80.43%)
TVS6_3	26809341	26292374	7.89G	97.19	92.5	52584748	43263917(82.27%)	43120911(82.0%)
TVS7_1	25685031	24880361	7.46G	96.6	91.5	49760722	40204719(80.8%)	40038671(80.46%)
TVS7_2	28874746	28232481	8.47G	96.29	90.95	56464962	45889190(81.27%)	45689173(80.92%)
TVS7_3	23380694	22383763	6.72G	97.2	92.65	44767526	36817462(82.24%)	36670967(81.91%)
TVS8_1	27453135	26750600	8.03G	96.2	90.85	53501200	43833110(81.93%)	43613489(81.52%)
TVS8_2	34022559	33069479	9.92G	96.88	92.18	66138958	54932687(83.06%)	54679524(82.67%)
TVS8_3	30945699	28689151	8.61G	96.86	92.1	57378302	47310837(82.45%)	47174743(82.22%)

A total of 19,737 genes were obtained from RNA-seq experiments of 24 samples. Among them, 7332 were novel genes predicted by stringtie software (Pertea et al., 2015). About 63.61% (12555/19737) of these genes were found to be expressed in at least one of the 24 samples (Supplementary Table 2). The number of expressed genes in different samples varied from 57.21% to 58.81%. About 0.56% - 0.88% of genes exhibited very high expression levels (FPKM > 1000) in different samples. The number of genes showing high (500 < FPKM ≤ 1000), moderate (10 < FPKM ≤ 500), low (0 < FPKM ≤ 10) and no (FPKM = 0) expression was similar in all samples (Supplementary Table 3). These analyses demonstrated sufficient coverage of the transcriptome during CF in *T*. *virens* GV29-8.

### Global Comparison of Transcriptomes Revealed Relationships Between Samples During CF

**FIGURE 2:**
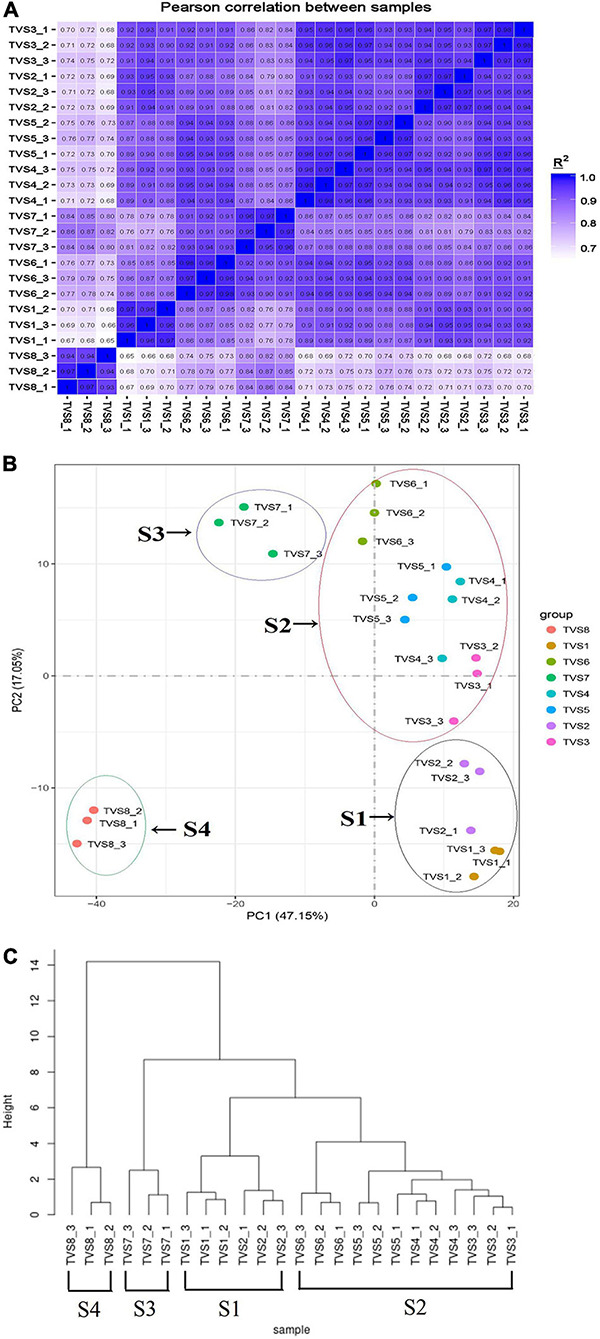
Correlation between the transcriptomes of different samples during CF of *T*. *virens* GV29-8. **(A)** Pearson correlation coefficient analysis of RNA-seq data from 8 sampling time points of *T*. *virens* GV29-8. **(B)** Principal component analysis (PCA) plot showing clustering of transcriptomes of different *T*. *virens* GV29-8 samples. The samples in black, red, purple and green circles represent S1, S2, S3, and S4 samples, respectively. **(C)** Phylogenetic analysis of sample correlation coefficients.

We performed correlation coefficient and principal component analyses (PCA) based on Pearson correlation coefficient analysis of average FPKM values for all expressed genes in all samples (Figures 2A,B). According to correlation coefficient analysis, we used the longest distance method to construct the phylogenetic tree for the samples (Figure 2C; Murtagh and Legendre, 2014). The Pearson correlation coefficients between biological replicates varied from 0.93 to 0.98, demonstrating the high quality of the replicates (Figure 2A). PCA analysis showed that the 8 sampling time points could be clearly assigned into 4 stages, referred to as S1, S2, S3 and S4 (Figure 2B). TVS1 and TVS2, shown by microscopic observation to be in the mycelial growth stage, were grouped into S1. TVS3, TVS4, TVS5 and TVS6 were assigned to group S2. TVS3 represented the transition stage from mycelium to chlamydospore, although microscopic observation showed TVS3 to be in the mycelial growth stage and TVS4 was the first time point at which CF could be observed. PCA results indicated that TVS3 was closer to TVS4. Generally speaking, while gene expression takes precedence over phenotype, it is possible that the genes related to CF were already being expressed in TVS3. About half of the mycelium tips had produced chlamydospores in TVS6. Therefore, samples of TVS3-TVS6 were divided into S2, and represented as the early and middle stages of CF. Samples at TVS7 and TVS8 were separated from each other and other time points. All mycelium tips and parts of the mycelium interior produced chlamydospores in TVS7. Mycelia autolysis was observed during TVS8. Therefore, TVS7 and TVS8 were classified into S3 and S4, respectively. S3 represents the flourishing stage, and S4 was the late stage of CF and initial mycelia autolysis. The results of phylogenetic analysis of sample correlation coefficients were consistent with those of the PCA analysis. This indicated that expression patterns in the mycelium growth stage (S1), early and middle stage of CF (S2), flourishing stage of CF (S3) and late stage of CF and mycelia initial autolysis (S4) were strikingly different from one another.

### Differential Gene Expression During CF

**FIGURE 3:**
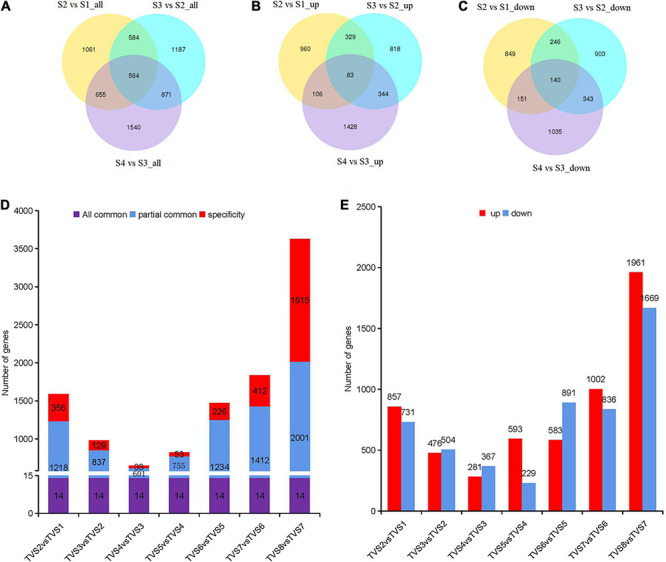
DEGs analysis among samples of adjacent stages and time points. Diagrams comparing shared and specific DEGs between adjacent developmental stages and sampling time points of CF. Venn diagrams of S2 vs S1, S3 vs S2 and S4 vs S3. **(A)** All DEGs. **(B)** Up-regulated DEGs. **(C)** Down-regulated DEGs. The non-overlapping regions represent DEGs unique to each comparison; Overlapping regions represent DEGs shared by two or more combinations. The number in the circle represents the number of DEGs. **(D)** Diagrams comparing shared and specific DEGs among samples of adjacent time points. The number of DEGs are indicated in the center of histograms. “All common” represents the DEGs that were shared by all comparisons. “Partially common” refers to DEGs that were shared by two or more comparisons. “Specific” refers to DEGs that were unique to a specific comparison. **(E)** Up-regulated and down-regulated DEG distribution between adjacent time points. The number of DEGs are indicated above the histograms.

According to PCA analysis, we divided the 8 sampling time points into 4 groups. A total of 6462 DEGs were obtained by comparison with adjacent stages (Supplementary Table 4). There were 2864, 3206 and 3630 DEGs screened from S2 vs S1, S3 vs S2 and S4 vs S3, respectively (Figure 3A). The largest number of up-regulated (1961) and down-regulated (1669) DEGs were contained in S4 vs S3 (Figures 3B,C). Reference genome annotation indicated that S2 vs S1, S3 vs S2, and S4 vs S3 contained 1689, 1761, and 2166 hypothetical protein genes and 500, 645, and 573 novel genes lacking annotation information, respectively. In order to better understand the function of these genes, NR, NT, Ko, Swiss, Pfam, GO and KOG database annotations were added in this study (Supplementary Table 4). Gene annotations showed that DEGs in S2 vs S1 were mainly related to metabolic and oxidation-reduction processes. In S3 vs S2, DEGs associated with oxidation-reduction, transcription, and transport processes dominated. In S4 vs S3, DEGs related to oxidation-reduction, transcription, and transport process were still activity (Supplementary Table 4).

Differentially expressed genes analysis for the 8 sampling time points was also performed by comparing adjacent time points (Figures 3D,E and Supplementary Table 5). 1588, 980, 648, 822, 1474, 1838 and 3630 DEGs were included in TVS2 vs TVS1, TVS3 vs TVS2, TVS4 vs TVS3, TVS5 vs TVS4, TVS6 vs TVS5, TVS7 vs TVS6 and TVS8 vs TVS7, respectively. There were 14 DEGs shared by the 7 comparisons. The greatest number of stage-specific DEGs were found in the TVS8 vs TVS7 (1615) comparison, while only 33 DEGs were unique to that of TVS4 vs TVS3 (Figure 3D and Supplementary Table 5). The largest number of up-regulated (1961) and down-regulated (1669) DEGs were identified in TVS8 vs TVS7 (Figure 3E and Supplementary Table 5).

### Functional Classification of DEGs

**FIGURE 4:**
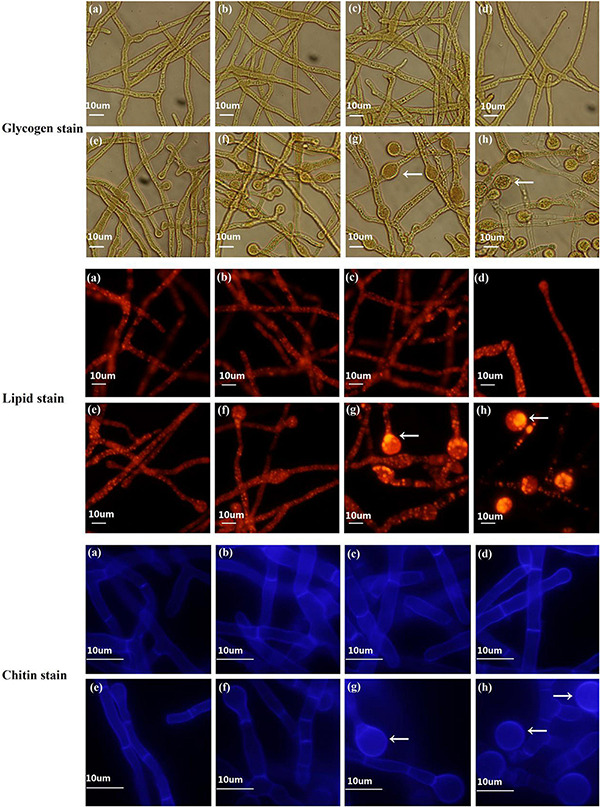
Fluorescence staining to determine dynamic changes in chlamydospore components in *T. virens* GV29-8. Glycogen, lipid, and chitin components were stained with KI and I_2,_ nile red and calcofluor white, respectively. **(a–h)** represent the samples of TVS1, TVS2, TVS3, TVS4, TVS5, TVS6, TVS7, and TVS8 of *T. virens* GV29-8, respectively. Scale bar = 10 μm.

All DEGs were classified into biological process (BP), cellular component (CC) and molecular function (MF) categories by comparison between samples from 4 adjacent stages (Supplementary Figure 1 and Supplementary Table 6). In S2 vs S1, organonitrogen compound metabolic (GO:1901564) was the most significantly enriched GO term in BP. 144 DEGs were down-regulated, while only 12 DEGs belonging to this term were up-regulated (Supplementary Table 7). Among them, 96 down-regulated DEGs were linked to organonitrogen compound biosynthetic processes, half of which were ribosomal proteins (e.g., ribosomal protein S2, TRIVIDRAFT_74494). Other down-regulated DEGs mainly corresponded with amino acid metabolism (e.g., aspartate-tRNA ligase, TRIVIDRAFT_79308; glutamine synthetase, TRIVIDRAFT_77167). Combined with microscopic observation of S1 and S2 (Figure 1), we speculated that organonitrogen compound metabolism might participate in induction of CF. It was reported that the oligopeptide transporter gene, *PTR2*, was significantly up-regulated under chlamydospore-inducing conditions in *C. albicans* (Palige et al., 2013). In this study, two *PTR2* genes (TRIVIDRAFT_50243 and TRIVIDRAFT_79497) were also differentially expressed during CF (Supplementary Table 2). In S3 vs S2, tetrapyrrole binding (GO:0046906) and heme binding (GO:0020037) were the most significantly enriched GO terms in MF. A total of 52 DEGs were recognized in these two GO terms. Among them, 16 and 36 DEGs were up-regulated and down-regulated, respectively. Most of the DEGs were cytochrome P450 family and antioxidants genes. For the up-regulated DEGs, 9 of them were P450 family genes. Some of these were homologous to genes involved in the synthesis of toxic secondary metabolites produced during fungal growth (e.g., O-methylsterigmatocystin oxidoreductase, TRIVIDRAFT_90820; versicolorin B desaturase, TRIVIDRAFT_212112), others were linked to the functions of detoxification and antioxidant activities (e.g., benzoate 4-monooxygenase, TRIVIDRAFT_47284; protein lutein deficient 5, TRIVIDRAFT_160585). Except for cytochrome P450 genes, the other up-regulated DGEs were mostly associated with antioxidant activity associated with ROS elimination (e.g., catalase, TRIVIDRAFT_38844/TRIVIDRAFT_45138). *Nox1* is a NADPH oxidase (Noxs) gene that produces ROS, regulates cell differentiation, and affects asexual spore formation in *T. atroviride* (Hernández-Oñate et al., 2012). In this study, *Nox1* (TRIVIDRAFT_32702) was differentially expressed during CF (Supplementary Table 2). For the down-regulated DEGs, 29 were P450 family genes, most of which were involved in the synthesis of toxic secondary metabolites (e.g., trichodiene oxygenase, TRIVIDRAFT_74291; abscisic acid 8′-hydroxylase, TRIVIDRAFT_216144/TRIVIDRAFT_230790; beta-amyrin 11-oxidase, TRIVIDRAFT_53690/TRIVIDRAFT_53375/TRIVIDR AFT_151337). Considering in S3 vs S2, DEGs were significantly enriched in secondary metabolic processes such as tetrapyrrole binding and heme binding, we speculated that secondary metabolic processes might further promote CF. During later stages (S4 vs S3), all significantly enriched GO terms were assigned into the BP category. Lipid metabolic process (GO:0006629) was the most significantly enriched GO term, in which 54 DEGs were enriched. These DEGs were mainly involved in phospholipid (e.g., ethanolaminephosphotransferase, novel.10349), glycerolipid (e.g., patatin-like phospholipase, TRIVIDRAFT_74421) and fatty acid (e.g., Sphingolipid C4-hydroxylase sur2, TRIVIDRAFT_43350) metabolic processes. It has been reported that mature chlamydospores contain a large number of fat droplets in *C. albicans* (Miller et al., 1974). In this study, Nile red staining showed that a large number of lipid droplets were formed during the late stages of CF (S3 and S4) (Figure 4), and genes related to fatty acid metabolism were indeed differentially expressed (Supplementary Table 23), including genes encoding acetyl-CoA C-acetyltransferase (TRIVIDRAFT_169943), enoyl-CoA hydratase (TRIVIDRAFT_87842), acetyl-CoA carboxylase (TRIVIDRAFT_78374), fatty acid synthase subunit beta, fungi type (TRIVIDRAFT_171412), fatty acid elongase 3 (TRIVIDRAFT_82162) and acetyl-CoA acyltransferase 1 (TRIVIDRAFT_80821). Genes related to lipid metabolism were differentially expressed in S4 and S3, suggesting that they might be associated with the CF.

Gene ontology analysis was also performed for the DEGs obtained from the comparisons between the 8 adjacent sampling time points (Supplementary Figure 2 and Supplementary Tables 8, 9). In TVS2 vs TVS1, the most significantly enriched GO term was protein metabolic process (GO:0019538) in BP. Small molecule metabolic process (GO:0044281) and organic acid metabolic process (GO:0006082) were the most significantly enriched GO terms in BP of TVS4 vs TVS3, and TVS6 vs TVS5, respectively. The most significantly enriched GO term in BP in TVS8 vs TVS7 was lipid metabolic process (GO:0006629). For the CC category, significantly enriched terms were similar in TVS3 vs TVS2, TVS4 vs TVS3, TVS5 vs TVS4 and TVS7 vs TVS6, comprised mainly of the integral component of membrane (GO:0016021) and intrinsic component of membrane (GO:0031224) categories. The MF category differed greatly across time point comparisons. The number of GO terms in the MF category was highest in TVS5 vs TVS4. Among these, purine ribonucleoside triphosphate binding (GO:0035639) was the most significantly represented GO term. Oxidoreductase activity, acting on CH-OH group of donors (GO:0016614) was significantly enriched in TVS7 vs TVS6.

The DEGs obtained by comparison across the 4 adjacent sampling stages were mapped to the KEGG database and tested for enrichment to further explore their functions (Supplementary Figure 3 and Supplementary Table 10). The ribosome (tre03010) pathway category was the most significantly enriched pathway in S2 vs S1. The DEGs in the ribosomal pathway were all ribosomal protein genes (e.g., ribosomal protein S2, TRIVIDRAFT_74494) and down regulated at S2. The metabolic pathway (tre01100) category was the most significantly enriched pathway in S3 vs S2 and S4 vs S3. The oxidative phosphorylation pathway (tre00190) always runs throughout the entire CF process, and all DEGs in this pathway were down-regulated. Additionally, the protein processing in endoplasmic reticulum (tre04141), tryptophan metabolism (tre00380) and N-glycan biosynthesis (tre00510) categories were also significantly enriched in S3 vs S2. There were 14 DEGs included in the N-glycan biosynthesis pathway. Among them, 10 were up-regulated and involved in mannan synthesis, including dolichyldiphosphatase (TRIVIDRAFT_25816), oligosaccharyltransferase complex subunit (TRIVIDRAFT_111960 and TRIVIDRAFT_121404), and dolichol-phosphate mannosyltransferase (TRIVIDRAFT_84289). 3 down-regulated DEGs (e.g., mannosyl-oligosaccharide alpha-1,2-mannosidase, TRIVIDRAFT_193426/TRIVIDRAFT_54636/TRIVIDRAFT_86 342) were involved in mannose degradation (Supplementary Tables 11, 22). Mannan is a common fungal cell wall component (Henry et al., 2016). Since DEGs related to the N-glycan biosynthesis pathway were significantly differentially expressed in S3 vs S2, we speculated that N-glycan biosynthesis might be involved in cell wall biosynthesis in chlamydospores.

KEGG enrichment analysis was also performed on DEGs obtained by comparison of across the 8 adjacent time points (Supplementary Figure 4 and Supplementary Table 12). The ribosome (tre03010) pathway was the most significantly enriched in TVS2 vs TVS1. The metabolic pathway (tre01100), with a *p*-value of nearly 0, was significantly enriched in TVS3 vs TVS2, TVS4 vs TVS3, TVS7 vs TVS6 and TVS8 vs TVS7. Glyoxylate and dicarboxylate metabolism (tre00630) was the next most enriched pathway in TVS3 vs TVS2. N-Glycan biosynthesis (tre00510) and various types of N-glycan biosynthesis (tre00513) were included in TVS7 vs TVS6. In TVS8 vs TVS7, the pentose phosphate pathway (tre00030), oxidative phosphorylation (tre00190) and ribosome biogenesis in eukaryotes (tre03008) categories were significantly enriched. The most abundant pathway for the TVS5 vs TVS4 and TVS6 vs TVS5 was cell cycle-yeast (tre04111). A total of 16 DEGs were contained in the cell cycle-yeast pathway, including genes encoding DNA replication licensing factor (TRIVIDRAFT_79120), structural maintenance of chromosome protein (TRIVIDRAFT_90905), and *CDC4* (TRIVIDRAFT_56502) (Supplementary Tables 13, 21). It was reported that *CDC10* or *CDC11* gene mutations lead to morphological defects of chlamydospores in *C. albicans* (Martin et al., 2005). In this study, CDC family genes (e.g., *CDC10*, TRIVIDRAFT_87191; CDC42; CDC48, TRIVIDRAFT_216898/TRIVIDRAFT_76254/TRIVIDRAFT_211309/TRIVIDRAFT_18 4509) were also differentially expressed during different CF stages. We deduced that the cell cycle pathway may be involved in regulating the mycelium to chlamydospore transformation. Fructose and mannose metabolism (tre00051) and the pentose phosphate pathway (tre00030) were also significantly enriched in TVS5 vs TVS4. For TVS6 vs TVS5, the meiosis-yeast (tre04113), nucleotide excision repair (tre03420) and DNA replication (tre03030) categories were significantly enriched (Supplementary Table 13).

### Clustering of Gene Expression Profiles Across the Eight Sampling Time Points

**FIGURE 5:**
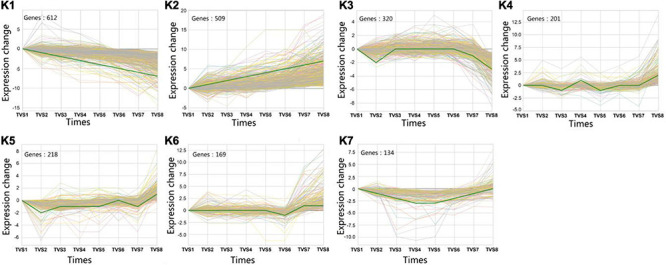
Series test of cluster (STC) analysis of DEGs between adjacent time points. STC analysis showing gene expression profiles. Seven clusters (K1-K7) were identified based on expression levels in 8 time points (TVS1, TVS2, TVS3, TVS4, TVS5, TVS6, TVS7, and TVS8).

A total of 5699 DEGs were obtained from 7 comparisons according to a *p*-value < 0.05 and | log_2_^(*FC*)^| > 0. Using STC analysis, 3581 DEGs were grouped into 50 clusters, which represent 62.8% of all DEGs (Supplementary Figure 5). Next, significance analysis of the DEG expression trends and fitting curves in the 50 clusters were performed. The results showed that DEG expression trends and fitting curves in 7 clusters (K1∼K7) were within the acceptable range (*p* < 0.05), while the remaining 43 clusters did not meet the significance standard (Figure 5 and Supplementary Table 14). Expression patterns of DEGs in the K1 cluster decreased from TVS1 to TVS8. However, DEGs in the K2 cluster showed an opposite expression pattern to those in the K1 cluster. Other DEGs from the K3, K4, K5, K6, and K7 clusters were predominantly expressed at one or more of the 8 time points.

**FIGURE 6:**
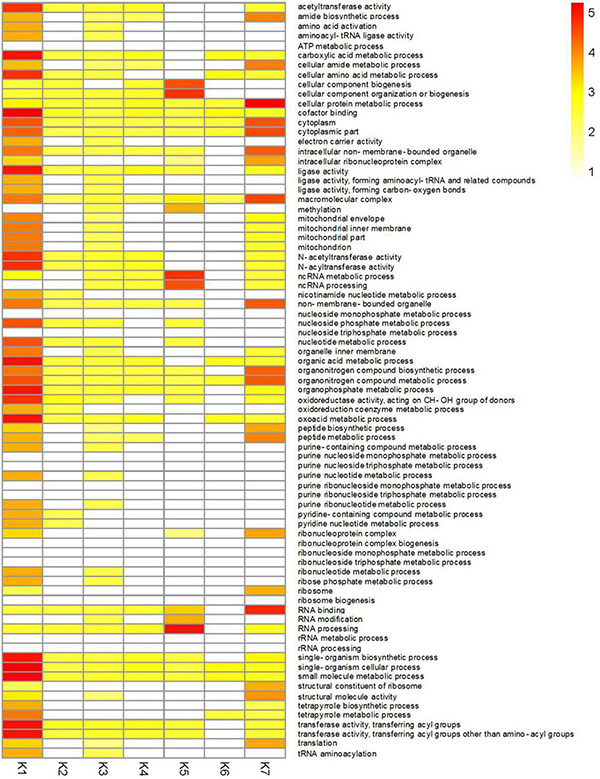
GO enrichment analysis of DEGs in different clusters of CF in *T. virens* GV29-8. Gene Ontology enrichment among the 7 clusters. Yellow to red: significant enrichment (*P* < 0.05). White, non-significant.

To better understand the functions of identified DEGs in different clusters, GO analysis was performed for each of the clusters (Figure 6 and Supplementary Table 15). The most enriched GO terms were found in the K1 cluster, which is consistent with this cluster having the most DEGs. Predominantly expressed DEGs in the K1 and K7 clusters had similar functional classifications, including organonitrogen compound biosynthetic process (GO:1901566), peptide biosynthetic process (GO:0043043), and the amide biosynthetic process (GO:0043604) categories. Furthermore, transferase activity and oxidoreductase activity metabolic processes were also significantly enriched in the K1 cluster.

### Construction of Gene Co-expression Networks

**FIGURE 7:**
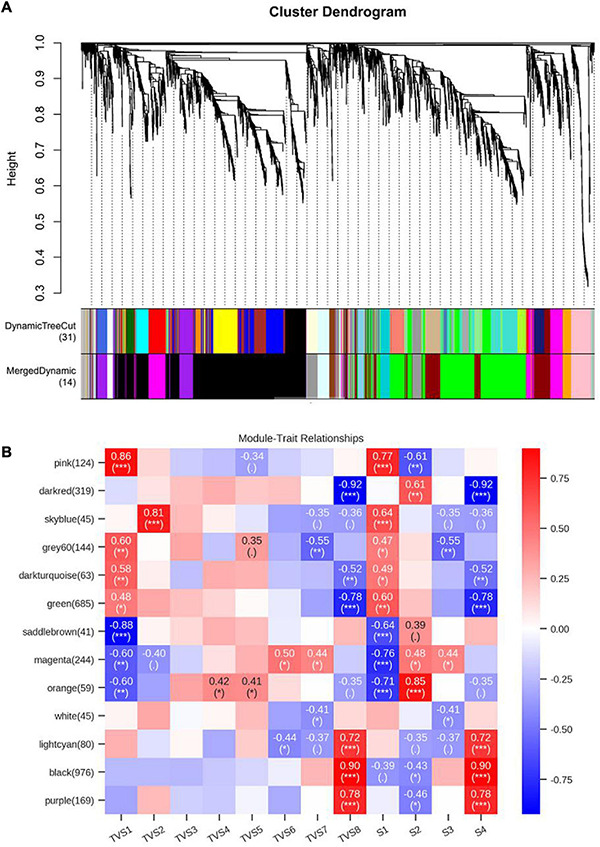
Weighted gene co-expression network analysis (WGCNA) of genes in different CF stages of *T. virens* GV29-8. **(A)** Cluster dendrogram of genes based on expression levels during the 8 sampling time points and 4 developmental stages. Each branch represents a gene and each color below represents a gene co-expression module. The dynamic tree cut indicates the modules were divided based on the gene clustering results. The merged dynamic cut indicates the modules were divided by combining modules with similar expression patterns. **(B)** Heatmap of gene expression patterns during the 8 sampling time points and 4 developmental stages. The expression patterns of 13 modules are shown by the heatmap. Each column represents a developmental stage. The module name is shown to the left side of each cell. The number of genes in each module is indicated in parentheses. Numbers in the table report the correlations of the corresponding module genes and stages, with the *p*-values (^∗^) printed below the correlations in parentheses. Each column corresponds to a specific stage. The scale bar on the right indicates the range of possible correlations from positive (red) to negative (blue).

Weighted gene co-expression network analysis can comprehensively reveal the relationships between gene expression and phenotype in the successive developmental stages (Langfelder and Horvath, 2008). Co-expression networks were constructed based on the previously reported methods (Guo et al., 2016; Riquelme Medina and Lubovac-Pilav, 2016). Each tree branch constitutes a module, and each leaf in the branch represents one gene, as shown in the hierarchical clustering tree (Figure 7A). For further analysis, we divided the tree from the resulting dendrogram into isolate modules. Ultimately, 14 modules (colored pink, dark red, sky blue, gray 60, dark turquoise, green, saddle brown, magenta, orange, white, light cyan, black, purple, and gray) were identified during the different CF stages. Genes in grey could not be assigned to any module, and bore no relative significance (Figure 7, Supplementary Tables 16, 17). The Pearson correlation algorithm was used to calculate the correlation coefficients and *p*-values of module characteristic genes and traits (Figure 7B). Genes which showed the most connectedness with other genes were identified as hub genes, as indicated by their high ImConn value (Eigengene connectivity). Correlation networks of different modules were constructed (Supplementary Figure 6). Each node represents a gene and the connecting lines (edges) between genes represent co-expression correlations (Langfelder and Horvath, 2008). WGCNA analysis showed that the pink (124) module genes exhibited high positive correlations with S1 (Figure 7B). Genes encoding ribosomal proteins comprised a large proportion of the pink module, including ribosomal protein S2 (TRIVIDRAFT_74494) and ribosomal protein L11 (TRIVIDRAFT_78230) (Supplementary Tables 16–18). KEGG enrichment analysis of genes in the pink module showed significant enrichment in the ribosome pathway (tre03010) (Supplementary Tables 19, 20). However, magenta (244) module genes exhibited high negative correlation with S1 (Figure 7B). Genes in the magenta module were mainly related to metabolic processes, including propionyl-CoA synthase (TRIVIDRAFT_55262) and L-threo-3-deoxy-hexylosonate aldolase (TRIVIDRAFT_215195) (Supplementary Tables 16–18). Magenta module genes were significantly enriched in the pentose and glucuronate interconversions (tre00040) and valine, leucine, and isoleucine degradation (tre00280) pathways (Supplementary Tables 19, 20). Orange module genes (59) were positively associated with S2 (Figure 7B). Genes related to oxidation-reduction (e.g., Sordaria macrospora k-hell, TRIVIDRAFT_46447) and transmembrane transport (e.g., major facilitator superfamily transporter, TRIVIDRAFT_43674) processes were included in the orange module (Supplementary Tables 16–18). KEGG enrichment analysis showed that genes in the orange module were mainly enriched in phenylalanine metabolism (tre00360), glutathione metabolism (tre00480), tryptophan metabolism (tre00380) and biosynthesis of secondary metabolites (tre01110) pathways (Supplementary Tables 19, 20). Black (976) and purple (169) modules exhibited high positive correlation with S4 (Figure 7B). Genes in the black module were mainly involved in metabolic, oxidation-reduction, and transport processes, including chitin synthase (TRIVIDRAFT_77496) and fatty acid synthase subunit beta fungi type (TRIVIDRAFT_171412) (Supplementary Tables 16–18). KEGG enrichment analysis showed that genes in these modules were all enriched in fatty acid metabolism (tre01212), biosynthesis of unsaturated fatty acids (tre01040), amino sugar and nucleotide sugar metabolism (tre00520), and starch and sucrose metabolism (tre00500) (Supplementary Tables 19, 20). Dark red (319) and green (685) modules genes were negatively correlated with S4 (Figure 7B). In these two modules, metabolic and oxidation-reduction genes were dominant. Enoyl-CoA hydratase (TRIVIDRAFT_87842), glucan endo-1,3-beta-D-glucosidase (TRIVIDRAFT_111476), glucanase (TRIVIDRAFT_89797) and phosphoglucomutase (TRIVIDRAFT_87728) were included (Supplementary Table 16, 17). The fatty acid degradation (tre00071) pathway was enriched in the dark red and green modules (Supplementary Tables 19, 20).

### Contents of Glycogen, Lipid, and Chitin Changes During CF in *T. virens* GV29-8

The dynamic changes of the components of CF were measured by staining. Microscopic observation showed that glycogen and lipid accumulation was noticeable in the interior of the mycelia at TVS1, TVS2 and TVS3, and chitin also accumulated in the cell wall and diaphragm of the mycelia at the same time. Chlamydospores were first observed at TVS4, a small amount of glycogen and lipids had accumulated in the center of chlamydospores, and chitin had accumulated in the chlamydospore cell wall by this time. After that, more glycogen and lipid in the cytoplasm and chitin in the chlamydospore cell wall and diaphragm had accumulated with the enlargement of chlamydospores. Glycogen, lipid and chitin accumulation was highest at S3 and S4 (Figure 4).

### The Chitin Synthase Gene (TRIVIDRAFT_90152) Is Essential for CF

It was reported that the amino sugar metabolism pathway was involved in CF of *Fusarium oxysporum* f. sp. *cubense* (FOC) (Ding et al., 2019). In the study, a large number of DEGs were enriched in the amino sugar and nucleotide sugar metabolism (tre00520) pathway. Among them, chitin synthase genes (*Chs*) TRIVIDRAFT_90152 had a large variation. Chitin synthases catalyze the formation of β-(1,4)-glycosidic bonds between GlcNAC residues to form the unbranched polysaccharide chitin, which is the major component of cell walls in most filamentous fungi (Liu et al., 2013). The chitin synthase genes (*Chs*) TRIVIDRAFT_90152 was significantly up-regulated by 5-fold from S1 to S4, and especially from S3 to S4 (Supplementary Table 25), which may promote the accumulation of chitin and contribute to the thickening of chlamydospore cell walls. In this study, chitin synthase gene (TRIVIDRAFT_90152) knockout mutant strains were constructed by using the split-PCR strategy (Supplementary Figure 7a). For mutant strains 1, 9 and 21, the PCR analysis confirmed the existence of the *hyg* sequence and the absence of the *Chs* sequence (Supplementary Figure 7b). Southern hybridization showed a single copy of the *Chs* sequence in wild-type *T. virens* GV29-8, and absence of the *Chs* sequence in the mutant strains (Supplementary Figure 7c). The results showed that the chitin synthase gene (TRIVIDRAFT_90152) of those 3 mutants (*Chs*Δ*1*, *Chs*Δ*9* and *Chs*Δ*21*) were successfully knocked out and selected for subsequent experiments.

**FIGURE 8:**
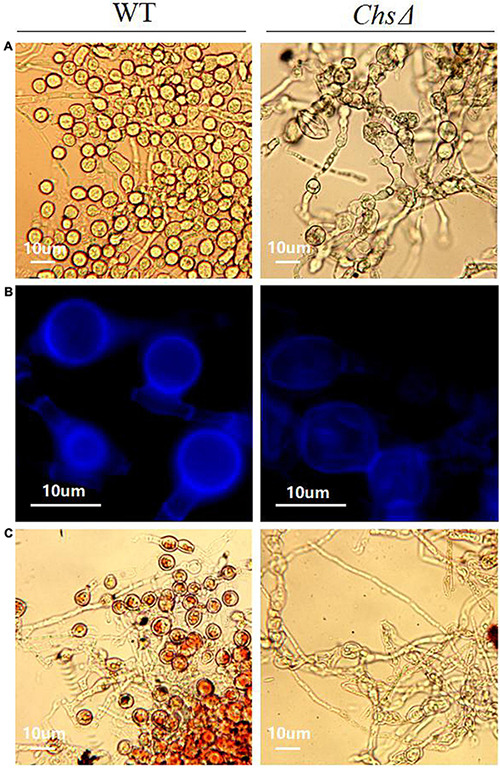
Effect of chitin synthase gene (TRIVIDRAFT_90152) deletion on CF in *T. virens* GV29-8. **(A)** The chlamydospores formed by the wild-type *T. virens* GV29-8 and chitin synthase gene (TRIVIDRAFT_90152) deletion mutant (*Chs*Δ). **(B)** Chitin staining of wild-type and *Chs*Δ mutant strains. **(C)** Oil red O staining of wild type and *Chs*Δ mutant strains.

The *Chs*Δ deletion mutants exhibited reduced mycelial growth, and produced significantly less mycelia than the wild-type *T. virens* GV29-8 on PDA medium (Supplementary Figure 7d). Sporulation structure of chlamydospore and mycelium morphology of wild-type *T. virens* GV29-8 and the chitin synthase gene (TRIVIDRAFT_90152) deletion mutant *Chs*Δ were observed by microscope; significant differences between the wild-type and *Chs*Δ mutant were observed. The results showed that chlamydospore formation was normal in the wild-type strain, while no chlamydospores were observed in the *Chs*Δ mutant, in which the mycelium was abnormally curved and twisted (Figure 8A). Fluorescence white staining showed that the chitin content in the *Chs*Δ mutant was significantly reduced and the chitin distribution was uniform in the mycelium cell wall (Figure 8B). Red oil O staining showed that no lipids accumulated in the *Chs*Δ mutant, which was a distinct structural feature of chlamydospores (Figure 8C). The results implied that the *Chs* gene plays a key role in CF. It is inferred that amino sugar and nucleotide sugar metabolism (tre00520) pathway affects the CF of *T. virens* GV29-8 strain.

### Validation of RNA-Seq-Based Gene Expression

**FIGURE 9:**
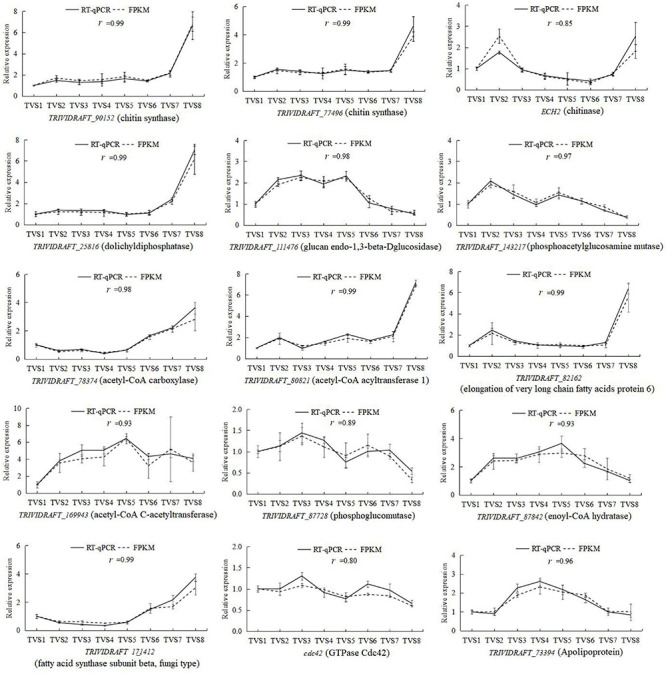
RT-qPCR determination of the expression levels of 15 selected DEGs during CF in *T. virens* GV29-8. The vertical axis represents the relative expression levels of DEGs across 8 sampling time points. The horizontal axis indicates the sampling time. Error bars represent the standard deviation of three independent replicates for each tested gene. The value *r* represents the correlation between RT-qPCR and RNA-seq data.

To validate the gene expression profiles obtained by RNA-Seq, reverse transcription quantitative PCR (RT-qPCR) was performed for 15 genes potentially related to CF. Correlation coefficients were calculated between the RNA-seq and RT-qPCR data for these 15 genes. The expression patterns of these genes determined by RT-qPCR were consistent with the RNA-Seq data (Figure 9), indicating the reliability and accuracy of the RNA-seq data.

## Discussion

Chlamydospore formation is a complex process regulated by many pathways. In this study, transcriptome sequencing was conducted for 8 developmental time points during the CF of *T. Virens* Gv29-8. Through transcriptomic data analysis, we found that organonitrogen deficiency, stress response and cell cycle regulation may be the main factors inducing CF. Additionally, large amounts of glycogen, lipids, chitin and mannose accumulated during the late stage of CF.

Pairwise comparisons were made for S1, S2, S3 and S4 stages. The S1 and S2 stages had the greatest differences in transcript and morphological changes during the process of developmental differentiation. DEGs were mostly significantly enriched in genes involved in organonitrogen compound metabolic processes (GO:1901564) and the ribosome pathway (tre03010) (Supplementary Figures 1, 3 and Supplementary Tables 6, 10). The majority of DEGs in the organonitrogen compound metabolic process and ribosome pathways were ribosomal protein genes, which were down-regulated in S2 (Supplementary Tables 7, 11). This suggests that organic nitrogen metabolism might be inhibited, protein biosynthesis was slowed down overall, and that nitrogen in the culture medium was relatively scarce in S2. In yeast, a poor nitrogen source (e.g., aminobutyric acid and urea) was only used when an abundant nitrogen source was scarce (Ramos et al., 1985; Marzluf, 1997; Palavecino et al., 2015). The 4-aminobutyrate transaminase (TRIVIDRAFT_57634) encoded by *GatA* catalyzed the conversion of aminobutyric acid into glutamic acid (Arst, 1976; Bailey et al., 1979), which was up-regulated from the latter of S1 (TVS2) to S4 in our study (Supplementary Table 2). This also indicates that available nitrogen may be scarce in the medium after the later stage of S1. The nitrogen source was an important factor affecting CF: even adding a small amount of peptone (0.2%) as an organic nitrogen source can strongly inhibit CF of *C. albicans* in culture (Böttcher et al., 2016). The nitrogen source mainly regulates CF via the TOR signaling pathway (Böttcher et al., 2016). Tor1 kinase is a highly conserved complex that senses the availability of nitrogen and other nutrients. Under conditions of sufficient nutrient availability, the activity of Tor1 is enhanced, which can drive the signal cascade that promotes cell proliferation (Kamada et al., 2004). Under nitrogen-poor conditions, it facilitates nuclear entry of the GATA factors, Gat1 and Gln3, to mediate expression of nitrogen catabolite repressed (NCR) genes (Beck and Hall, 1999; Cardenas et al., 1999). Mutations in either the *gat1* or *gln3* gene could inhibit CF (Böttcher et al., 2016). In our study, the seryl-tRNA synthetase encoding gene (TRIVIDRAFT_172396) which is involved in positive regulation of the TOR signaling pathway, and the nitrogen metabolism repressor gene (TRIVIDRAFT_176019), were down-regulated from S2 to S4 (Supplementary Table 2). The results showed that the availability of an abundant nitrogen source in the medium was reduced, thus the strain might be entering a nitrogen starvation state by S2 and subsequent stages. *PTR2* is a nitrogen starvation response gene, and a conserved high affinity transporter shown to physically interact with the nutrient-sensing *Tor1* and *Tor2* complexes in *Saccharomyces cerevisiae* (Aronova et al., 2007). Palige et al. (2013) showed that expression of the nitrogen starvation response gene *PTR2* was significantly up-regulated during the induction of CF in *C. albicans*. In our study, two *PTR2* genes, TRIVIDRAFT_50243 and TRIVIDRAFT_79497, were up-regulated and highly expressed in TVS2-TVS5 and TVS6-TVS8, respectively (Supplementary Table 2). Phenotypic results showed that chlamydospores initially formed on the tips of the mycelia at the early stage of S2. Subsequently, a large number of chlamydospores formed in the interior of mycelia at the later stages of S2-S4 (Figure 1). These two genes might play an important role in CF, and up-regulation of them at different periods might be related to regulation of different stages of CF. In summary, the strains made extensive use of the supplied nitrogen source in the early vegetative growth stage, leading to organic nitrogen deficiency in the late stage of S1, and the strains might induce CF by regulating differential expression of nitrogen utilization regulators.

The GO analysis showed that DEGs were most significantly enriched for tetrapyrrole binding (GO:0046906) and heme binding (GO:0020037) in S3 vs S2 (Supplementary Figure 1 and Supplementary Table 6). The DEGs in these 2 terms were mainly the cytochrome P450 family and antioxidant genes. Most of the P450 genes were homologous to genes required for synthesizing toxic secondary metabolites during the growth of fungi; others were linked to the function of detoxification and antioxidant (Supplementary Table 7). The results showed that not only nutrient deficiency, but also secondary metabolism and stress responses, may induce CF. Sclerotia, like chlamydospores, are also dormancy structures produced by many fungi. Studies showed that genes linked to secondary metabolite production also control sclerotia production (Jirjis and Moore, 1976; Willetts and Bullock, 1992; Kües, 2000; Georgiou et al., 2006; Bayram and Braus, 2012; Calvo and Cary, 2015; Song, 2018; Shu et al., 2019). For example, different members of the velvet protein family interact with each other and the non-velvet protein *LaeA*. *LaeA* is a methyltransferase-domain protein that functions as a regulator of secondary metabolism and sclerotial morphogenesis (Bayram and Braus, 2012; Calvo and Cary, 2015). Moreover, Mukherjee and Kenerley (2010) reported that the *Velvet1* gene mutation resulted in increased chlamydospore production under conditions of nutritional stress in *T. virens*. In this study, 2 new genes (novel.11798 and novel.972) were annotated as velvet factors and differentially expressed during CF (Supplementary Table 2). The P450 genes O-methylsterigmatocystin oxidoreductase and versicolorin B desaturase are mainly involved in the synthesis of aflatoxins in fungi. Aflatoxin is a highly toxic polyketone-derived secondary metabolite which can poison fungal cells. It was reported that the genes involved in aflatoxin synthesis are co-regulated with sclerotia formation in *Aspergillus flavus* (Chang et al., 2002; Duran et al., 2007; Cary et al., 2012; Calvo and Cary, 2015). Microsclerotia are another type of dormancy structure of many fungi, so we speculate the up-regulation of the O-methylsterigmatocystin oxidoreductase and versicolorin B desaturase genes might lead to the accumulation of aflatoxins and affect CF in *Trichoderma* (Supplementary Table 7). Benzoate 4-monooxygenase is a benzoate detoxification enzyme (Podobnik et al., 2008). Benzoic acid interferes with the permeability of microbial cells, inhibits the absorption of amino acids by the cell membrane and the activity of the cellular respiratory enzyme system, and hinders the condensation reaction of acetyl-COA to affect normal life activities of cells (Kresnowati et al., 2008). Up-regulated expression of the benzoate 4-monooxygenase gene could accelerate the catalytic conversion of benzoic acid to p-hydroxybenzoic acid in S3 (Supplementary Table 7), reducing harm to cells. It has been reported that a gene encoding the lutein deficient 5 protein was involved in biosynthesis of lutein in plants (Fiore et al., 2006; Kim and DellaPenna, 2006; Kim et al., 2009). In this study, TRIVIDRAFT_160585 was annotated as a protein lutein deficient 5 homologous gene, which was up-regulated from S2 to S4 (Supplementary Table 7), possibly accelerated biosynthesis of lutein. Lutein exhibits strong anti-oxidant capabilities and can reduce the damage of reactive oxygen species (ROS) to cells (Picot et al., 2013). ROS are formed in during normal life activities of cells; excessive ROS accumulation can directly or indirectly damage cellular components (e.g., DNA, proteins and lipids), and lead to cell death (Jamieson, 1998; Ruhland and Reniere, 2019). *Nox1* is a NADPH oxidase (Noxs) family gene that participates in the production of ROS, which can regulate cell differentiation and affect the formation of asexual spores in *T. atroviride* (Hernández-Oñate et al., 2012). In our study, up-regulation of the *Nox1* gene might promote the production of ROS in S2 stage (Supplementary Table 2). The expression levels of ROS scavenging genes were relatively lower during S1 and S2, which might lead to the accumulation of ROS, placing the strain under an environment of oxidative stress, further promoting the transformation of mycelia into chlamydospores. Conversely, to survive, genes encoding catalase were up-regulated at S3 and S4 to eliminate ROS and alleviate damage to cells caused by oxidative stress (Supplementary Table 7). Most of the down-regulated DEGs were P450 family genes and involved in the synthesis of toxic secondary metabolites (Supplementary Table 7). Trichodiene oxygenase, is involved in the synthesis of trichodermin, a highly effective antifungal substance (Takahashi-Ando et al., 2008; Malmierca et al., 2012). TRIVIDRAFT_74291 was annotated as homologous genes of the Trichodiene oxygenase gene, in this study. In plants, abscisic acid 8′-hydroxylase takes part in the synthesis of abscisic acid (Saito et al., 2004; Kushiro et al., 2004; Umezawa et al., 2006). In our study, TRIVIDRAFT_230790 and TRIVIDRAFT_216144 were annotated as being homologous with the abscisic acid 8′-hydroxylase gene and might be involved in the formation of abscisic acid (Supplementary Table 7). Excessive accumulation of abscisic acid may promote transformation of mycelia into dormant spores. Beta-amyrin 11-oxidase is the precursor substance used to synthesize glycyrrhizin in plants (Seki et al., 2008). TRIVIDRAFT_53690, TRIVIDRAFT_53375 and TRIVIDRAFT_151337 were annotated as homologous genes of the beta-amyrin 11-oxidase gene (Supplementary Table 7). By comparing the expression levels of these genes at the S1, S2, S3 and S4, we found that most of the DEGs involved in toxic secondary substance synthesis were highly expressed during S1 and S2, and lowly expressed during S3 and S4. DEGs encoding detoxifying enzyme and free radical scavenging enzymes were highly expressed during S3 and S4 (Supplementary Table 7). This suggests that the rapid growth of mycelia under abundant nutrient conditions would be accompanied by the accumulation of toxic and harmful secondary metabolites. When large amounts of harmful substances accumulated, strains will produce detoxifying and free radical scavenging enzymes to alleviate adverse stress. Alternatively, fungi can change their developmental strategies in favor of long-term survival by producing resistant propagules. Therefore, we hold the opinion that both nutrient decline in the media, and non-nutrient factors such as environmental conditions and secondary stress factors caused by metabolism, are involved in fungi CF.

Cell differentiation might be a vital factor in regulating the transformation of mycelia into chlamydospores. KEGG enrichment analysis showed that cell cycle-yeast (tre04111) genes were significantly enriched in TVS5 vs TVS4 and TVS6 vs TVS5 (Supplementary Figures 4, 8 and Supplementary Tables 12, 13). DEGs in this pathway were involved in different phases of cell differentiation. It has been reported that the formation of chlamydospores is regulated by different cell cycle genes in *C. albicans*, and deletion of the *CDC10* or *CDC11* genes lead to morphological defects in chlamydospores (Martin et al., 2005). The *CDC10* gene was highly expressed during S1 and S2, then significantly down-regulated in S3 and S4 (Supplementary Table 2). Higher expression of the *CDC10* gene in the first two stages may positively regulate CF. Additionally, the *CDC4* gene was also significantly up-regulated, 5.66-fold, from S2 (TVS4) to S4 (TVS8) (Supplementary Table 21). Other CDC family genes (CDC42 and CDC48) were also differentially expressed during different stages (Supplementary Table 2). Microsclerotia are a type of dormant structure, which is considered to originate from polar hyphae in fungi (Biles et al., 1992; Colotelo, 1974; Jackson and Jaronski, 2009; Yin et al., 2012). Inhibition of Rho/Rac/CDC42 family GTPases have been noted to significantly reduce polarized hyphal growth and microsclerotia production (Jiang et al., 2014; Song et al., 2016). Similarly, we deduced that the GTPase *CDC42* gene was highly expressed in S1∼S3 and might be related to CF. According to the above data, we hypothesize that DEGs in cell cycle pathways may be involved in regulating CF.

N-glycan biosynthesis pathway (tre00510) genes, which are involved in both anabolism and catabolism of mannan, were also significantly enriched in S3 vs S2 (Supplementary Figures 3, 9 and Supplementary Tables 10, 11). Mannan is a primary component of cell walls in *C. albicans*, and deletion of the α-1,6-mannosyltransferase gene can affect cell wall integrity (Pérez-García et al., 2016). Damage to the mannose synthesis pathway prevents CF in *C. albicans* (Whelan et al., 1990). This suggests that the N-glycan biosynthesis pathway may be related to cell wall construction of chlamydospores in *Trichoderma*. There were 14 DEGs in the N-glycan biosynthesis pathway, 10 of which are involved in mannan synthesis and up-regulated at S3 (Supplementary Figure 9 and Supplementary Tables 11, 22). For example, dolichyldiphosphatase catalyzes dolichyl diphosphate into dolichol phosphate, the first step of the N-glycan synthesis reaction (Janik et al., 2019). The gene encoding dolichyldiphosphatase was significantly up-regulated at S3. Three DEGs encoding mannosyl-oligosaccharide alpha-1,2-mannosidase, which is involved in mannose degradation, were all down regulated at S3 (Supplementary Figure 9 and Supplementary Tables 11, 22). It could be inferred that up-regulation of mannan anabolism genes and down-regulation of catabolism genes could lead to mannan accumulation during S3, which might affect chlamydospore cell wall formation.

We found that lipid metabolism genes were significantly enriched in S4 vs S3, and DEGs in this category were mainly correlated with fat, phospholipid, and sterol metabolism (Supplementary Figure 1 and Supplementary Table 7). It has been reported that the mature chlamydospore center of *C. albicans* contains a large number of fat droplets (Miller et al., 1974). The main components of fats are glycerol and fatty acids. In our study, DEGs in all 3 comparisons (S2 vs S1, S3 vs S2 and S4 vs S3) appeared to be enriched for pathways related to fatty acid metabolism (including fatty acid metabolism (tre01212), fatty acid degradation (tre00071) and biosynthesis of unsaturated fatty acids (tre01040), and the number and expression level of DEGs changed at different stages (Supplementary Table 10). A total of 20 DEGs were enriched in these 3 pathways. Among them, 6 genes were involved in the fatty acid degradation pathway, and the others were all related to fatty acid synthesis (Supplementary Figure 10 and Supplementary Table 23). By analyzing the gene expression levels of these 20 DEGs in S1∼S4, we found that DEGs involved in the fatty acid degradation pathway were highly expressed in S1 and S2, then decreased in S3 and S4. For example, the genes encoding acetyl-CoA C-acetyltransferase and enoyl-CoA hydratase were up-regulated 2.9 and 6.4-fold from S1 to S2, respectively, and then significantly down-regulated in S3 and S4. The expression of acetyl-CoA C-acetyltransferase gene was down-regulated 15-fold from S2 to S4. In contrast, most DEGs in the fatty acid synthesis pathway maintained lower expression levels from S1 to the middle stages of S2 (TVS1∼TVS5), then were significantly up-regulated from late S2 to S4 (TVS6 ∼ TVS8). For example, the acetyl-CoA carboxylase gene increased 5.96-fold from the early stage of S2 (TVS4) to S4 (TVS8). Acetyl-CoA carboxylase is a rate-limiting enzyme of fatty acid synthesis, which can catalyze acetyl-CoA into malonyl-CoA (Wakil et al., 1958; Davis et al., 2000; Tong, 2013); up-regulated expression of this gene during CF (S2∼S4/TVS4∼TVS8) may accelerate the accumulation of fatty acids. The gene *Fas1*, encoding the fatty acid synthase subunit beta was up-regulated 5.8-fold from the late stage of S2 (TVS5) to S4. Fatty acid synthetase catalyzes the formation of long-chain fatty acids from acetyl-CoA, malonyl-CoA and NADPH (Kuziora et al., 1983; Nguyen et al., 2009). *FAS1* mutations reduced lipid deposition in conidia of *Magnaporthe oryzae* (Sangappillai and Nadarajah, 2020). Additionally, genes encoding fatty acid elongase 3 and acetyl-CoA acyltransferase 1 are involved in fatty acid synthesis and were significantly up-regulated at S4 (Supplementary Figure 10 and Supplementary Table 23). This indicated that fatty acid degradation may provide energy for basic life functions during the mycelium growth and early CF stages. Biosynthesis of fatty acid pathway genes were significantly up-regulated at the later stage of CF, which could promote fat accumulation in chlamydospores. Nile red staining results also confirmed that a large number of lipid droplets formed in the late stage (S3 and S4) of CF (Figure 4). Chlamydospores are propagules produced under unfavorable conditions, and the accumulation of lipids could provide energy for spore germination once more favorable environmental conditions are re-established.

To obtain a comprehensive understanding of which genes contribute to different the developmental stages of CF, WGCNA was performed on samples from the 8 time points and 4 stages (Figure 7; Supplementary Tables 16, 17). Genes with similar expression patterns likely had similar functions, so they were grouped into the same module. Pearson correlation coefficients were used to judge the relationship between different modules and phenotypes at different development stages. Our results show that the pink and magenta modules were highly positively and negatively correlated with S1, respectively (Figure 7). The genes in the pink module were significantly enriched in the ribosome (tre03010) pathway (Supplementary Table 19), which was consistent with the results of previous KEGG enrichment analyses at S1 (Supplementary Figure 3 and Supplementary Table 10). Genes in magenta are mainly enriched in the pentose and glucuronate interconversions (tre00040) and valine, leucine, and isoleucine degradation (tre00280) pathways (Supplementary Table 19). Pentose and glucuronate interconversions is one of the primary pathways involved in carbohydrate utilization in fungal strain (Cui et al., 2020). These results show that the strain absorbed nutrients in the medium and synthesized various substances to meet its own growth needs at S1, and as nutrients became depleted in the late growth stage, nutrient assimilation was reduced. The orange module showed high positive correlation with S2 (Figure 7), in which glutathione metabolism (tre00480), phenylalanine metabolism (tre00360) and tryptophan metabolism (tre00380) related genes were enriched (Supplementary Table 19). The products of these pathways have confirmed roles in antioxidant, antifungal and antibacterial activity (Allen et al., 1960; Haile and Dekebo, 2013). These results and previous GO analysis of DEGs in S3 vs S2 were consistent with each other (Supplementary Figure 1 and Supplementary Table 6). We speculate that the culture environment became hostile at S2, stimulating the fungal strain to produce resistant substances and dormancy structures in favor of survival. Black, purple, and light cyan modules showed high positive correlation with S4, while dark red and green modules showed high negative correlation (Figure 7). Genes in black and purple modules were enriched in the fatty acid metabolism (tre01212) and biosynthesis of unsaturated fatty acids (tre01040) pathways. Conversely, genes in the dark red and green modules were enriched in fatty acid metabolism (tre01212) and fatty acid degradation (tre00071) pathways (Supplementary Table 19). This suggests that fatty acid synthesis was dominant at S4 and may contribute to accumulation of fatty acids, which is consistent with the results of GO and KEGG enrichment analyses in S4 vs S3. Additionally, starch and sucrose metabolism (tre00500) and amino sugar and nucleotide sugar metabolism (tre00520) pathways were also enriched in these 4 modules (Supplementary Table 19). Starch and sucrose metabolic pathways were mainly related to anabolism and catabolism of glycogen. In *Coprinopsis cinerea*, glycogen served as a carbon storage molecule, and was actively transformed between monomeric and polymeric forms during morphogenesis (Jirjis and Moore, 1976). The gene encoding glucan endo-1,3-beta-D-glucosidase is relevant to the process of glycogen decomposition, which transforms Udp-glucose into D-glucose. The gene was significantly up-regulated at S1 and S2, then declined sharply at S3 and S4, after which it remained at a relatively low expression level (Supplementary Figure 11 and Supplementary Table 24). Phosphoglucomutase mediates the conversion of α-D-Glucose 6-phosphate and α-D-Glucose 1-phosphate, which is involved in glycogen decomposition (Ray and Roscelli, 1964; Mesak and Dahl, 2000). Similarly, the gene expression level was also significantly reduced at S4 (Supplementary Figure 11 and Supplementary Table 24). The down-regulation of glucan endo-1,3-beta-D-glucosidase and phosphoglucomutase genes may be conducive to glycogen accumulation at S3 and S4, which is consistent with the results of I_2_ and KI staining which showed a large amount of glycogen accumulated in chlamydospores at S3 and S4 (Figure 4). Therefore, mycelia growth accompanied a gradual decrease in nutrient availability in the medium at the early stage of CF (S1 and S2), leading to nutritional stress, after which glycogen decomposition increased to alleviate the nutritional stress. However, this did not prevent CF, due to regulation of nutrient consumption, stress caused by secondary metabolites, and induction of the cell cycle. A sharp decrease in glycogen decomposition at the later stage of CF may be conducive to glycogen accumulation in chlamydospores, which provided energy for germination under more favorable conditions. Previous studies showed that large amounts of glycogen accumulated in the cytoplasm of chlamydospores to store energy (Son et al., 2012). Chitin and glucan are the main components of the cell wall in fungal chlamydospores (Roncero et al., 2016). Chlamydospores have a large cell volume and thick cell walls. Therefore, large amounts of glucan and chitin might be synthesized during the formation of mature chlamydospores. Excluding genes related to glycogen metabolism, expression of genes encoding glucanase and alpha, alpha-trehalase (TRIVIDRAFT_42275) also sharply declined at S4 (Supplementary Tables 19, 24), resulting in accumulation of glucan and trehalose and thickening of chlamydospore cell walls, which helps fungal cells overcome osmotic stress. Fungi, through acetylation, convert glucose into N-acetyl-D-glucosamine (GlcNAC) and synthesize chitin by chitin synthase. Chitin synthases and deacetylase might play a key role in the construction of chlamydospore cell walls. There were 12 genes involved in amino sugar and nucleotide sugar metabolism, contained in the black, purple, dark red, and green modules, which correlated with S4 (Figure 7 and Supplementary Figure 12; Supplementary Tables 19, 25). It was reported that GlcNAC inhibited CF of *Fusarium oxysporum* f. sp. *cubense* (Ding et al., 2019). Chitinase (*Ech2*), involved in the amino sugar and nucleotide sugar metabolism pathway, could break down chitin into GlcNAC (Tzelepis et al., 2012). In our study, the Chitinase (*Ech2*) gene was significantly down-regulated and maintained a low expression level from S1 to S2, then experienced a slow increase until S3 and S4 (Supplementary Table 25). This suggests that low expression of *Ech2* at S2 might reduce the accumulation of GlcNAC and promote the formation of chlamydospores. Increased expression of the *Ech2* gene during S3 and S4 (Supplementary Table 25), may play a role in the aging of mycelia and cell-wall degradation, releasing the internal nutrients and providing nutrients and energy for CF when medium nutrients become lacking. Additionally, the chitin synthase genes (TRIVIDRAFT_77496 and TRIVIDRAFT_90152) was significantly up-regulated (3.1 and 5-fold) from S1 to S4, especially from S3 to S4 (Supplementary Table 25), which may promote the accumulation of chitin and contribute to the thickening of chlamydospore cell walls. In our study, deletion of the chitin synthase gene TRIVIDRAFT_90152 blocked chitin biosynthesis of the cell wall, resulting in mycelium dysplasia and an inability to form normal chlamydospores in *T. virens* GV29-8 (Figure 8). This indicated that the chitin synthase gene (TRIVIDRAFT_90152) plays an important role in cell wall formation of mycelia and was necessary for CF. The amino acid sugar and nucleotide sugar metabolism (tre00520) pathway affects the CF of *T. virens* GV29-8 strain.

In conclusion, CF is a complex biological process that acts via multiple integrated signal transduction pathways. In the early stage, fungal strains were mainly engaging in assimilation and absorption of nutrients in the medium for mycelium growth. When nutrients in the medium were depleted to a certain threshold, the stress stimulated the fungal strain to induce chlamydospore formation. Simultaneously, the organism’s own life activities led to changes in the culture environment conditions and the production of secondary metabolites and ROS, which further stimulated chlamydospore production. Pathway associated with the cell cycle were differentially expressed to regulate cell differentiation of the fungal strain. Finally, through the synergistic action of multiple pathways and genes, necessary components of chlamydospore structure, such as glycogen, lipids, and thickened cell walls, were synthesized to guarantee the formation of chlamydospores. Our results provide insights into the key pathways and hub genes involved in CF, from a transcriptomic perspective. Further verification through deletion of these genes is needed to clarify their functions in CF. This is the first detailed developmental transcriptomic study of CF in *T. virens* GV29-8. which will aid in further understanding of the mechanisms underlying CF in fungi and provides a foundation for improving the production and utilization of chlamydospore containing agents.

## Data Availability Statement

The original contributions presented in the study are publicly available. This data can be found here: NCBI repository, https://www.ncbi.nlm.nih.gov/assembly/GCA_000170995.2.

## Author Contributions

XP and BW contributed the experiment and writing for the manuscript. SZ did the experiment. ML and XJ contributed the design of the experiments and writing for the manuscript. All authors contributed to the article and approved the submitted version.

## Conflict of Interest

The authors declare that the research was conducted in the absence of any commercial or financial relationships that could be construed as a potential conflict of interest.

## Footnotes

1 https://www.ncbi.nlm.nih.gov/genome/?term=GCA_000170995.2

## Supplementary Material

Supplementary Figure 1 Gene ontology (GO) functional classification of DEGs among samples of adjacent stages. **(a)**, **(b)**, and **(c)** represent functional classification of DEGs between S2 vs S1, S3 vs S2, and S4 vs S3, respectively. The bars represent significantly enriched GO terms (*p* < 0.05), and the numbers beside terms indicate the number of DEGs for each term. The numbers in the centers of red and blue bars represent the number of up-regulated and down-regulated DEGs in the term, respectively. Because the picture is too big, we split it into 2 parts to display it more clearly.

Supplementary Figure 2 GO functional classification of DEGs between adjacent sampling time points. **(a), (b), (c), (d), (e), (f)** and **(g)** represent GO functional classification of DEGs between TVS2 vs TVS1, TVS3 vs TVS2, TVS4 vs TVS3, TVS5 vs TVS4, TVS6 vs TVS5, TVS7 vs TVS6 and TVS8 vs TVS7, respectively. The bars represented significantly enriched GO terms (*p* < 0.05), and the number beside each term indicates the number of DEGs in the term. The numbers in the centers of the red and blue bars represent the number of up-regulated and down-regulated DEGs in the term, respectively. Because the picture is too big, we split it into 2 parts to display it more clearly.

Supplementary Figure 3 Kyoto encyclopedia of genes and genomes (KEGG) functional classification of DEGs between adjacent stages. The bars represent significantly enriched KEGG pathways (*p* < 0.05), and the number beside the term indicates the number of DEGs included in the pathway. The numbers in the centers of red and blue bars represents up-regulated and down-regulated DEGs, respectively.

Supplementary Figure 4 KEGG functional classification of DEGs among samples of adjacent time points. The bars represent significantly enriched KEGG pathways (*p* < 0.05), and the number beside the term indicates the number of DEGs included in the pathway. The numbers in the centers of red and blue bars represents up-regulated and down-regulated DEGs, respectively.

Supplementary Figure 5 Summary Plot of all DEGs STC classification. black_Hub_network. dark red_Hub_network. darkturquoise_Hub_network. green_Hub_network. grey60_Hub_network. light cyan_Hub_network. magenta_Hub_network. orange_Hub_network. pink_Hub_network. purple_Hub_network. saddle brown_Hub_network. sky blue_Hub_network. white_Hub_network.

Supplementary Figure 6 Protein-protein interaction (PPI) network of genes in the black, dark red, dark turquoise, green, grey60, light cyan, magenta, orange, pink, purple, saddle brown, sky blue and white modules.

Supplementary Figure 7 Construction of chitin synthase gene (TRIVIDRAFT_90152) deletion mutants and the effect on phenotypes of the *T. virens* GV29-8. **(a)** Chitin synthase gene (TRIVIDRAFT_90152) deleting strategy. The *Chs* gene of *T.virens* GV29-8 was knocked out by split-PCR strategy. The split-marker cassettes were developed using fusion PCR, and involved two rounds of PCR. First round PCR: the upstream and downstream homologous fragments of *Chs* were amplified using primers upF/upR and downF/downR with the genome DNA of *T.virens* GV29-8 as template, respectively. Fragment of the hygromycin gene was amplified with the plasmid pKH-KO as template, and *hyg*F/*hyg*R as primer. Second round PCR: the upstream homologous fragment of *Chs* gene and the *hyg* gene fragment from the first round of PCR products were simultaneously used as templates, and the final fragment (1) was amplified by using the primer up2F/*hyg*2R, which contained the upstream homologous region of the *Chs* gene and two thirds of the hygromycin gene fragment. Similarly, the downstream homologous region of the *Chs* gene and two thirds of the hygromycin gene fragment was amplified (2). There were 554 bp *hyg* gene overlap region between (1) and (2) in the center of *hyg*. **(b)** PCR identification of *Chs* and *hyg* genes in *Chs*Δ*1*, *Chs*Δ*9*, *Chs*Δ*21* and WT. M was a 250-bp ladder. PCR templates were genomic DNA of *Chs*Δ*1*, *Chs*Δ*9*, *Chs*Δ*21* and wild-type GV29-8 respectively. **(c)** Southern hybridization analysis. Probe: *Chs*, DNAs of wild-type GV29-8, *Chs*Δ*1*, *Chs*Δ*9*, and *Chs*Δ*21* were digested by BamHI/NotI. (d) Phenotypes of the wild-type *T. virens* GV29-8 and *Chs*Δ mutant strains.

Supplementary Figure 8 Cell cycle-yeast pathway (tre04111). DEGs in cell cycle-yeast pathway: Apc/c: anaphase-promoting complex subunit 8 (25794716); CDC45: cell division control protein 45 (25786744); MCM: DNA replication licensing factor MCM3 (25797744/25797702/25797864); CDC7: cell division control protein 7 (25795105); Pom1: dual specificity tyrosine-phosphorylation-regulated kinase (25791399); Met30: F-box and WD-40 domain protein MET30 (25795627); Smc2: structural maintenance of chromosome 2 (25797454); Smc4: structural maintenance of chromosome 4 (25798962); Mcm2: DNA replication licensing factor MCM2 (25797864); Mcm6: DNA replication licensing factor MCM6 (25797744); Mcm7: DNA replication licensing factor MCM7 (25797702); Orc1: origin recognition complex subunit 1 (25793997); Orc6: origin recognition complex subunit 6 (25787414); Mcm4: DNA replication licensing factor MCM4 (25798055). The green box represents a specific gene or enzyme in *Trichoderma Reesei* and does not indicate up-regulation or down-regulation of the gene.

Supplementary Figure 9 N-Glycan biosynthesis pathway (tre00510). DEGs in N-Glycan biosynthesis pathway: 3.6.1.43: dolichyldiphosphatase (25792540); DMP1: dolichol-phosphate mannosyltransferase (25798517); ALG5: dolichyl-phosphate beta-glucosyltransferase (25795393); OST: oligosaccharyltransferase complex subunit alpha (25786852/25787193/25798795); MAN1: mannosyl-oligosaccharide alpha-1,2-mannosidase (25798667/25789754/25795445); STT: dolichyl-diphosphooligosaccharide-protein glycosyltransferase (25797749); ALG1: beta-1,4-mannosyltransferase (25795163); GCS1: mannosyl-oligosaccharide glucosidase (25793447); DPM3: dolichol-phosphate mannosyltransferase subunit 3 (25799030), beta-1,4-N-acetylglucosaminyltransferase (25793326). The green box represents a specific gene or enzyme in *Trichoderma Reesei* and does not indicate up-regulation or down-regulation of the gene. DEGs in fatty acid degradation pathway: 4.2.1.17: enoyl-CoA hydratase (25798760); 2.3.1.9: acetyl-CoA C-acetyltransferase (25788734/25797542); 5.3.3.8: Delta3-Delta2-enoyl-CoA isomerase (25795747); 1.1.1.1: alcohol dehydrogenase, propanol-preferring (25793381); 1.3.8.7: acyl-CoA dehydrogenase (25788983). DEGs in biosynthesis of unsaturated fatty acid pathway: ① 2.3.1.199: fatty acid elongase 3 (25798319/25794969);②1.1.1.62/1.1.1.330: 17 beta-estradiol 17-dehydrogenase/very-long-chain 3-oxoacyl-CoA reductase (25797972/25798045/25792427); ③ 4.2.1.134: very-long-chain (3R)-3-hydroxyacyl-CoA dehydratase (25796723); ④ 1.3.1.93: very-long-chain enoyl-CoA reductase (25792467); ⑤ 2.3.1.16: acetyl-CoA acyltransferase 1 (25798206/25786723). DEGs in fatty acid biosynthesis pathway: 6.4.1.2: acetyl-CoA carboxylase (25798001); 2.3.1.86: fatty acid synthase subunit beta, fungi type (25788772); 2.3.1.179: 3-oxoacyl-[acyl-carrier-protein] synthase II (25798380); 1.1.1.100: 3-oxoacyl-[acyl-carrier protein] reductase (25792427); 6.2.1.3: long-chain acyl-CoA synthetase (25798208).

Supplementary Figure 10 Fatty acid metabolic pathway(fatty acid degradation(tre00071), biosynthesis of unsaturated fatty acid (tre01040) and fatty acid biosynthesis pathway (tre00061)). The green box represents a specific gene or enzyme in *Trichoderma Reesei* and does not indicate up-regulation or down-regulation of the gene.

Supplementary Figure 11 Starch and sucrose metabolism pathway (tre00500). DEGs in starch and sucrose metabolism pathway: 3.2.1.39: glucan endo-1,3-beta-D-glucosidase (25786810); 3.2.1.21: beta-glucosidase (25797680); 3.2.1.28: alpha, alpha-trehalase (25794082); 5.4.2.2: phosphoglucomutase (25798750). The green box represents a specific gene or enzyme in *Trichoderma Reesei* and does not indicate up-regulation or down-regulation of the gene.

Supplementary Figure 12 Amino sugar and nucleotide sugar metabolism (tre00520). DEGs in Amino sugar and nucleotide sugar metabolism pathway: 3.2.1.14: chitinase (25795194/25786774/25786843); 3.2.1.52: beta-N-acetylhexosaminidase (25786802); 5.4.2.3: phosphoacetylglucosamine mutase (25787876); 3.2.1.165: exo-1,4-beta-D-glucosaminidase (25794739); 2.4.1.16: chitin synthase (25798917/25797926/25797171); 2.3.1.4: glucosamine-phosphate N-acetyltransferase (25797997); 5.4.2.2: phosphoglucomutase (25798750). The green box represents a specific gene or enzyme in *Trichoderma Reesei* and does not indicate up-regulation or down-regulation of the gene.
