# Supplementary material for: Transcriptome Dynamics Underlying Chlamydospore Formation in Trichoderma virens GV29-8
Source: Front Microbiol. 2021 Jun 8;12:654855. doi: 10.3389/fmicb.2021.654855 (PMC8217873; doi:10.3389/fmicb.2021.654855)
Supplement: Supplementary Figure 1 — Gene ontology (GO) functional classification of DEGs among samples of adjacent stages. (a), (b), and (c) represent functional classification of DEGs between S2 vs S1, S3 vs S2, and S4 vs S3, respectively. The bars represent significantly enriched GO terms (p < 0.05), and the numbers beside terms indicate the number of DEGs for each term. The numbers in the centers of red and blue bars represent the number of up-regulated and down-regulated DEGs in the term, respectively. Because the picture is too big, we split it into 2 parts to display it more clearly. [file Data_Sheet_1.pdf]

(a)

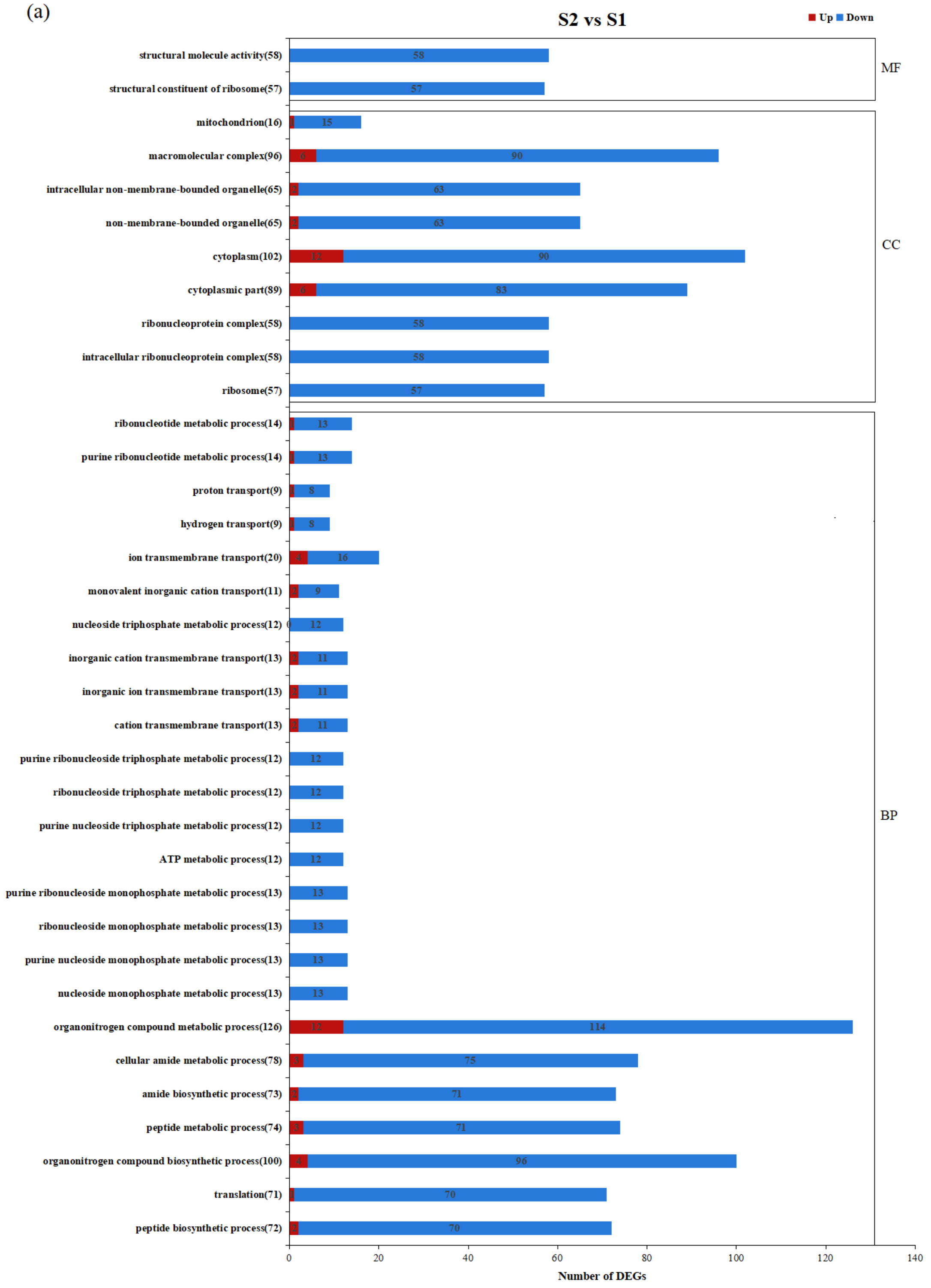

S2 vs S1
Up
Down
structural molecule activity(58)
structural constituent of ribosome(57)
mitochondrion(16)
macromolecular complex(96)
intracellular non-membrane-bounded organelle(65)
non-membrane-bounded organelle(65)
cytoplasm(102)
cytoplasmic part(89)
ribonucleoprotein complex(58)
intracellular ribonucleoprotein complex(58)
ribosome(57)
ribonucleotide metabolic process(14)
purine ribonucleotide metabolic process(14)
proton transport(9)
hydrogen transport(9)
ion transmembrane transport(20)
monovalent inorganic cation transport(11)
nucleoside triphosphate metabolic process(12)
inorganic cation transmembrane transport(13)
inorganic ion transmembrane transport(13)
cation transmembrane transport(13)
purine ribonucleoside triphosphate metabolic process(12)
ribonucleoside triphosphate metabolic process(12)
purine nucleoside triphosphate metabolic process(12)
ATP metabolic process(12)
purine ribonucleoside monophosphate metabolic process(13)
ribonucleoside monophosphate metabolic process(13)
purine nucleoside monophosphate metabolic process(13)
nucleoside monophosphate metabolic process(13)
organonitrogen compound metabolic process(126)
cellular amide metabolic process(78)
amide biosynthetic process(73)
peptide metabolic process(74)
organonitrogen compound biosynthetic process(100)
translation(71)
peptide biosynthetic process(72)
MF
CC
BP
0
20
40
60
80
100
120
140
Number of DEGs

(b)

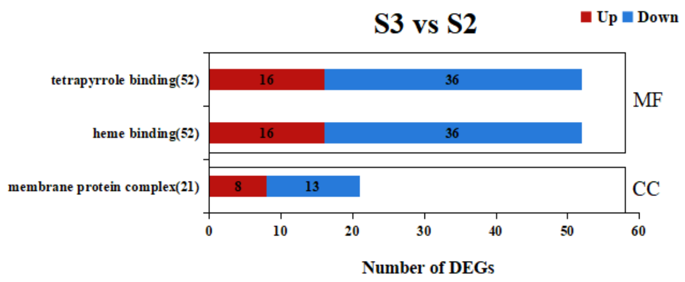

(c)

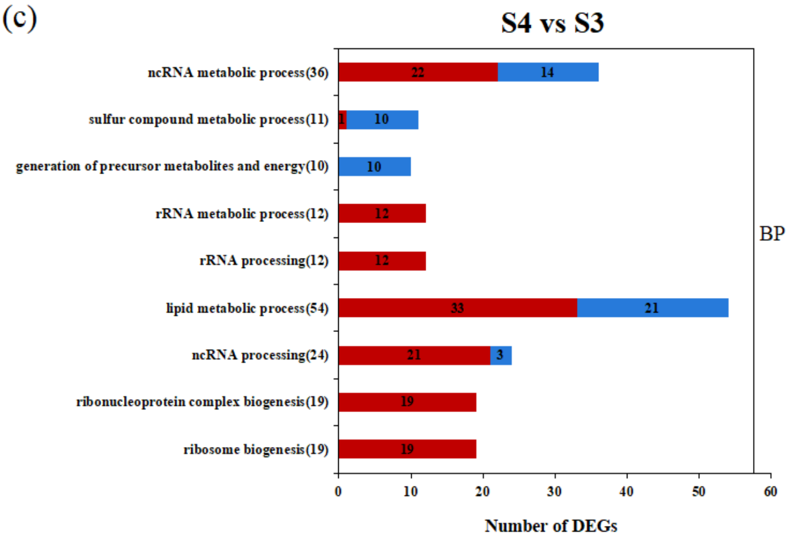

Fig. S1 GO functional classification of DEGs among samples of adjacent stages. (a), (b) and (c) represent functional classification of DEGs between S2 vs S1, S3 vs S2 and S4 vs S3, respectively. The bars represent significantly enriched GO terms ($P < 0.05$), and the numbers beside terms indicates the number of DEGs for each term. The number in the center of red and blue bars represents the number of up-regulated and down-regulated DEGs in the term, respectively. Because the picture is too big, we splited it into 2 parts to see it more clearly.

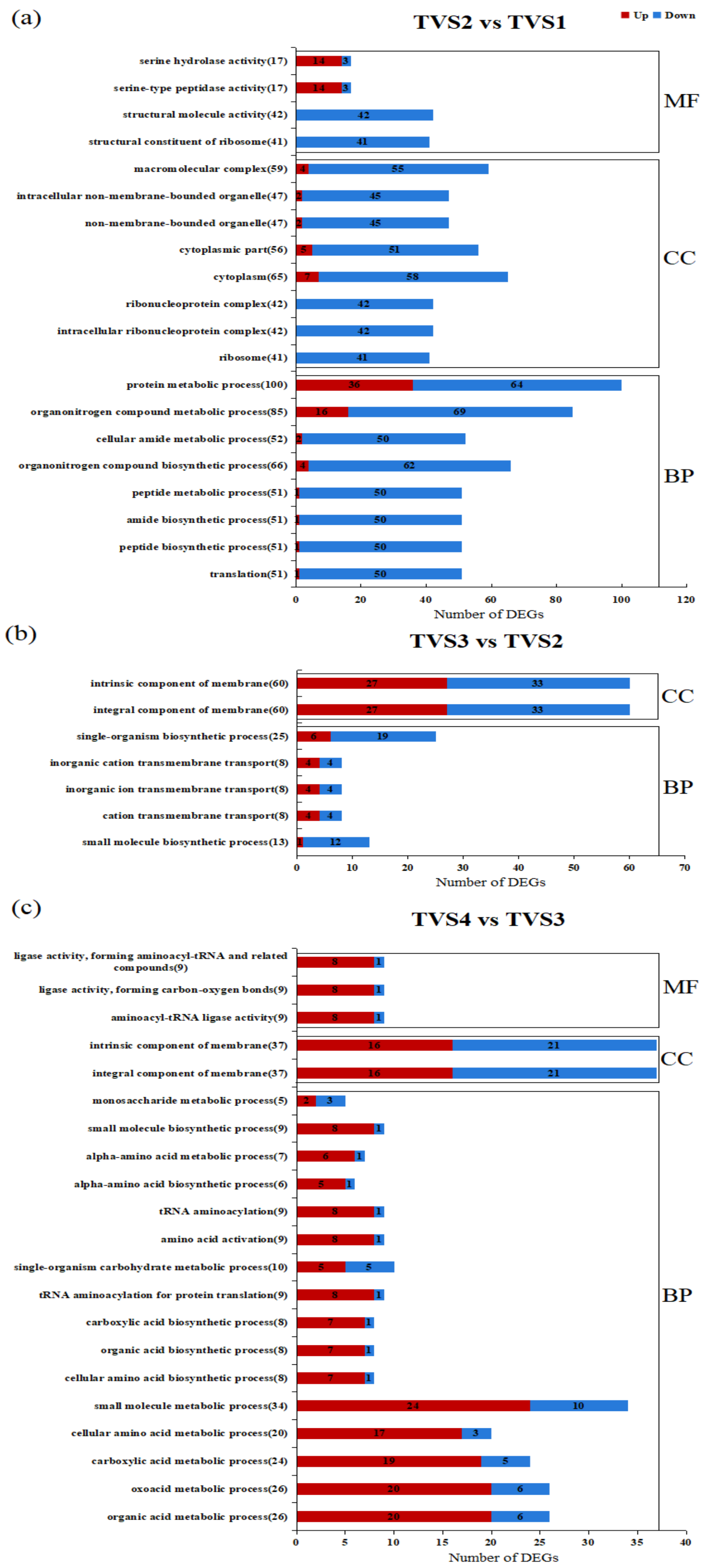
(a)
TVS2 vs TVS1
Up Down
serine hydrolase activity(17) 14 3
serine-type peptidase activity(17) 14 3
structural molecule activity(42) 42
structural constituent of ribosome(41) 41
macromolecular complex(59) 4 55
intracellular non-membrane-bounded organelle(47) 2 45
non-membrane-bounded organelle(47) 2 45
cytoplasmic part(56) 5 51
cytoplasm(65) 7 58
ribonucleoprotein complex(42) 42
intracellular ribonucleoprotein complex(42) 42
ribosome(41) 41
protein metabolic process(100) 36 64
organonitrogen compound metabolic process(85) 16 69
cellular amide metabolic process(52) 2 50
organonitrogen compound biosynthetic process(66) 4 62
peptide metabolic process(51) 1 50
amide biosynthetic process(51) 1 50
peptide biosynthetic process(51) 1 50
translation(51) 1 50
MF
CC
BP
0 20 40 60 80 100 120
Number of DEGs
(b)
TVS3 vs TVS2
intrinsic component of membrane(60) 27 33
integral component of membrane(60) 27 33
single-organism biosynthetic process(25) 6 19
inorganic cation transmembrane transport(8) 4 4
inorganic ion transmembrane transport(8) 4 4
cation transmembrane transport(8) 4 4
small molecule biosynthetic process(13) 1 12
CC
BP
0 10 20 30 40 50 60 70
Number of DEGs
(c)
TVS4 vs TVS3
ligase activity, forming aminoacyl-tRNA and related compounds(9) 8 1
ligase activity, forming carbon-oxygen bonds(9) 8 1
aminoacyl-tRNA ligase activity(9) 8 1
intrinsic component of membrane(37) 16 21
integral component of membrane(37) 16 21
monosaccharide metabolic process(5) 2 3
small molecule biosynthetic process(9) 8 1
alpha-amino acid metabolic process(7) 6 1
alpha-amino acid biosynthetic process(6) 5 1
tRNA aminoacylation(9) 8 1
amino acid activation(9) 8 1
single-organism carbohydrate metabolic process(10) 5 5
tRNA aminoacylation for protein translation(9) 8 1
carboxylic acid biosynthetic process(8) 7 1
organic acid biosynthetic process(8) 7 1
cellular amino acid biosynthetic process(8) 7 1
small molecule metabolic process(34) 24 10
cellular amino acid metabolic process(20) 17 3
carboxylic acid metabolic process(24) 19 5
oxoacid metabolic process(26) 20 6
organic acid metabolic process(26) 20 6
MF
CC
BP
0 5 10 15 20 25 30 35 40
Number of DEGs

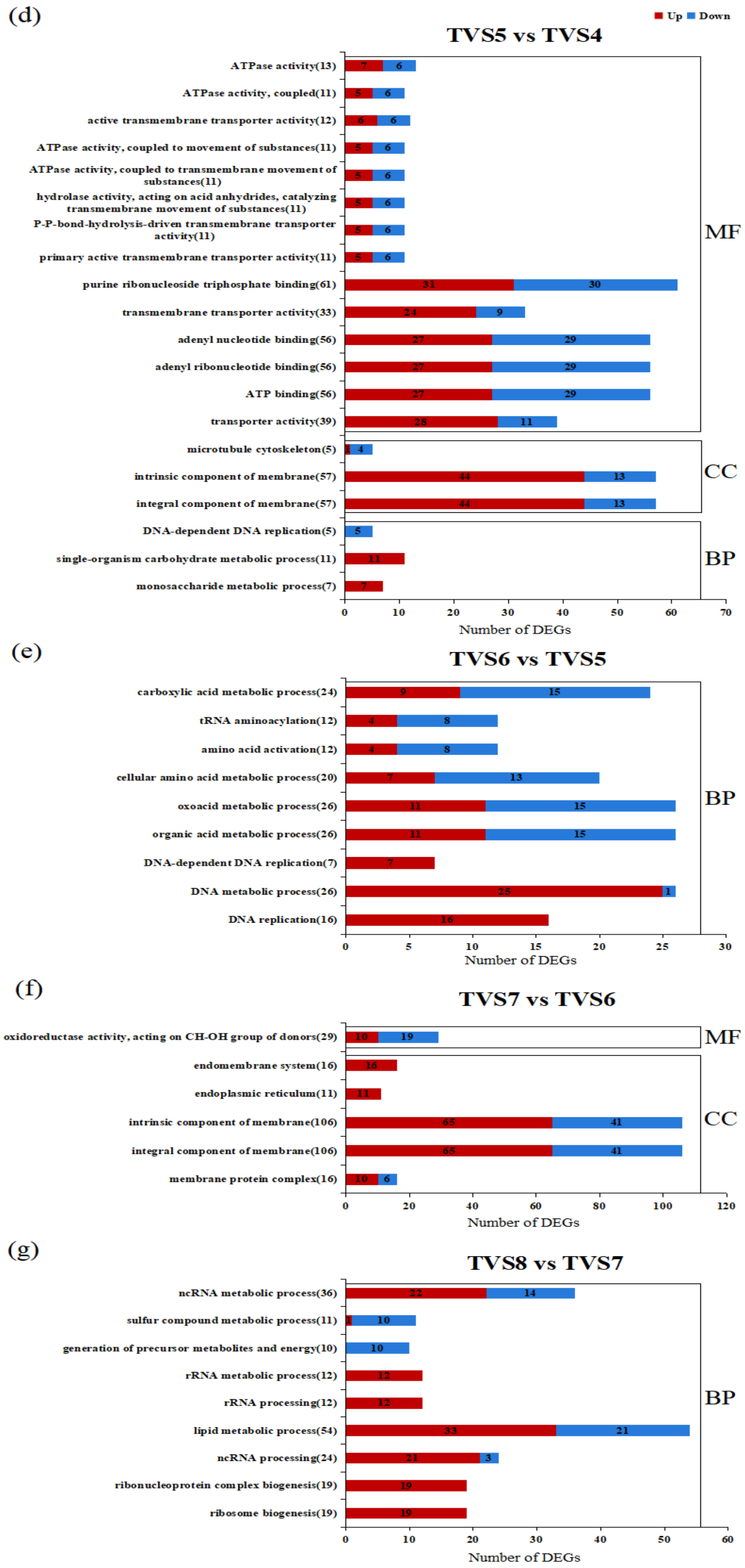
(d)
Up Down
TVS5 vs TVS4
ATPase activity(13)
ATPase activity, coupled(11)
active transmembrane transporter activity(12)
ATPase activity, coupled to movement of substances(11)
ATPase activity, coupled to transmembrane movement of substances(11)
hydrolase activity, acting on acid anhydrides, catalyzing transmembrane movement of substances(11)
P-P-bond-hydrolysis-driven transmembrane transporter activity(11)
primary active transmembrane transporter activity(11)
purine ribonucleoside triphosphate binding(61)
transmembrane transporter activity(33)
adenyl nucleotide binding(56)
adenyl ribonucleotide binding(56)
ATP binding(56)
transporter activity(39)
microtubule cytoskeleton(5)
intrinsic component of membrane(57)
integral component of membrane(57)
DNA-dependent DNA replication(5)
single-organism carbohydrate metabolic process(11)
monosaccharide metabolic process(7)
MF
CC
BP
Number of DEGs
(e)
TVS6 vs TVS5
carboxylic acid metabolic process(24)
tRNA aminoacylation(12)
amino acid activation(12)
cellular amino acid metabolic process(20)
oxoacid metabolic process(26)
organic acid metabolic process(26)
DNA-dependent DNA replication(7)
DNA metabolic process(26)
DNA replication(16)
BP
Number of DEGs
(f)
TVS7 vs TVS6
oxidoreductase activity, acting on CH-OH group of donors(29)
endomembrane system(16)
endoplasmic reticulum(11)
intrinsic component of membrane(106)
integral component of membrane(106)
membrane protein complex(16)
MF
CC
Number of DEGs
(g)
TVS8 vs TVS7
ncRNA metabolic process(36)
sulfur compound metabolic process(11)
generation of precursor metabolites and energy(10)
rRNA metabolic process(12)
rRNA processing(12)
lipid metabolic process(54)
ncRNA processing(24)
ribonucleoprotein complex biogenesis(19)
ribosome biogenesis(19)
BP
Number of DEGs

Fig. S2 GO functional classification of DEGs between adjacent sampling time points. (a), (b), (c), (d), (e), (f) and (g) represent GO functional classification of DEGs between TVS2 vs TVS1, TVS3 vs TVS2, TVS4 vs TVS3, TVS5 vs TVS4, TVS6 vs TVS5, TVS7 vs TVS6 and TVS8 vs TVS7, respectively.The bars represented significantly enriched GO terms ($P < 0.05$), and the number beside each term indicates the number of DEGs in the term. The number in the center of the red and blue bars represents the number of up-regulated and down-regulated DEGs in the term, respectively. Because the picture is too big, we splited it into 2 parts to see it more clearly.

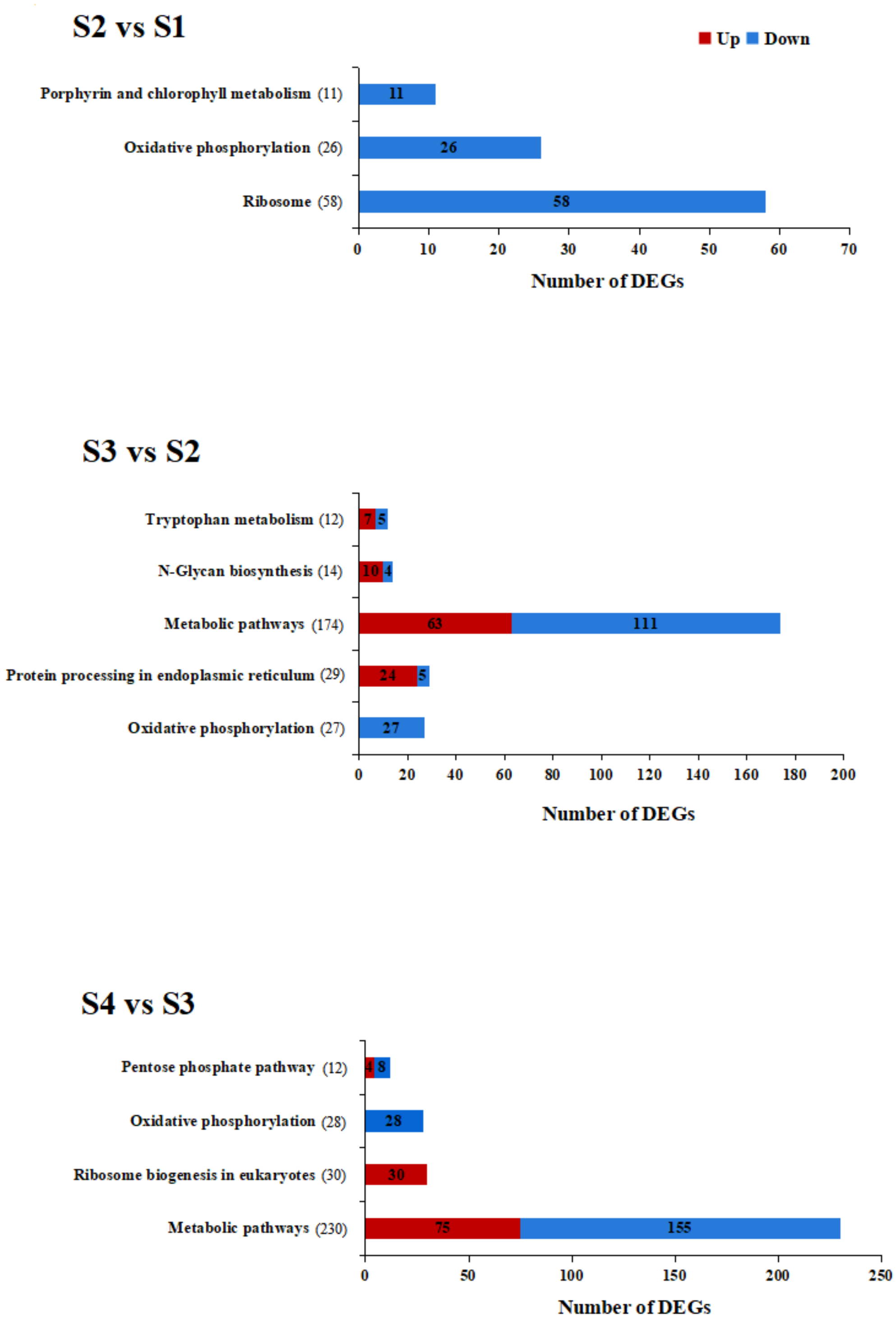

Fig. S3 KEGG functional classification of DEGs between adjacent stages. The bars represent significantly enriched KEGG pathways ($P < 0.05$), and the number beside the term indicates the number of DEGs included in the pathway. The number in the center of red and blue bars represents up-regulated and down-regulated DEGs, respectively.

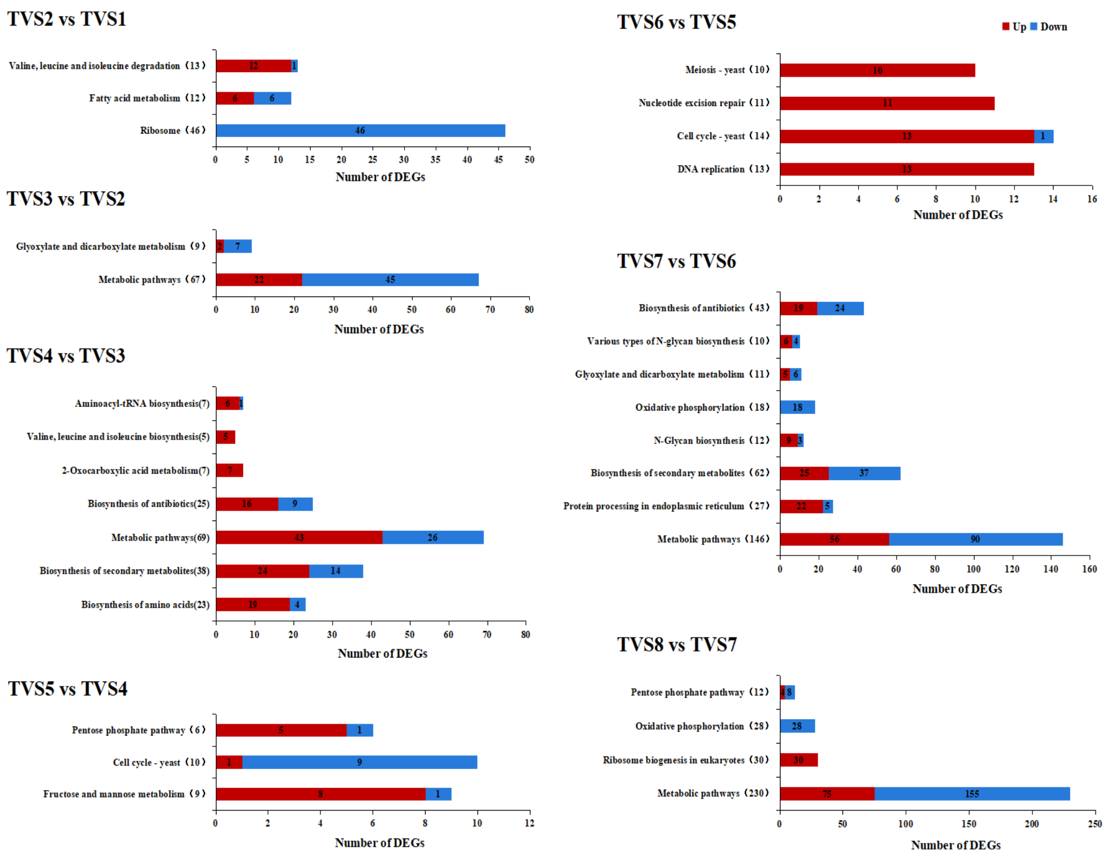

Fig. S4 KEGG functional classification of DEGs among the samples of adjacent time points. The bars represent significantly enriched KEGG pathways ($P < 0.05$), and the number beside the term indicates the number of DEGs included in the pathway. The number in the center of red and blue bars represents up-regulated and down-regulated DEGs, respectively.

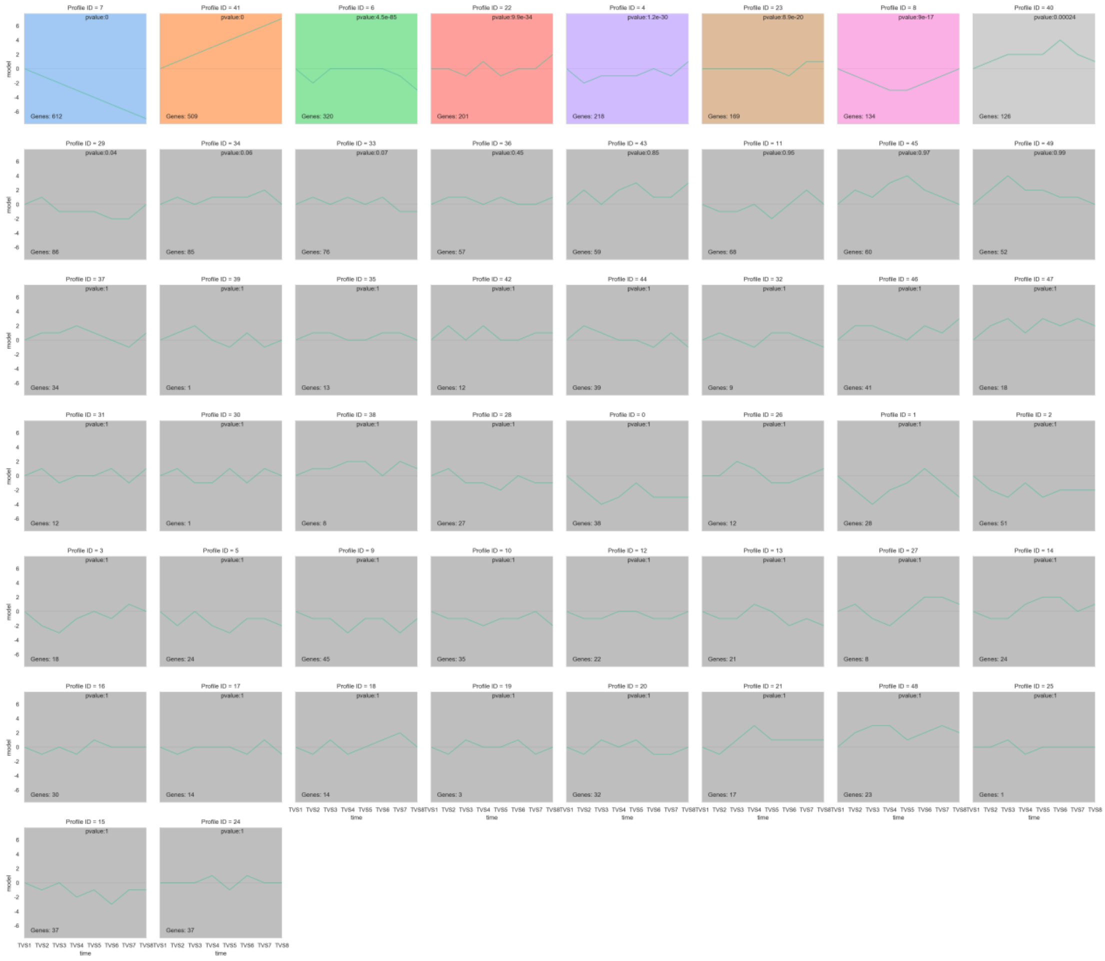

Fig. S5 Summary Plot of all DEGs STC classification.

black_Hub_network

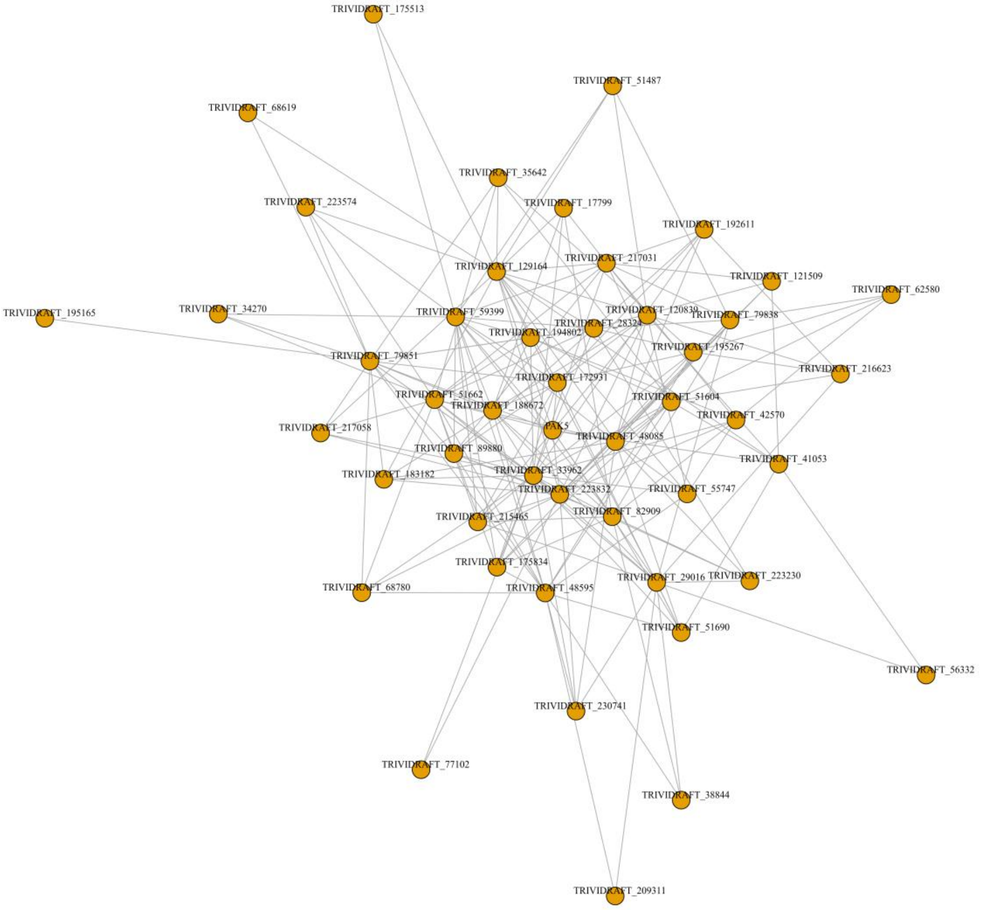

dark red_Hub_network

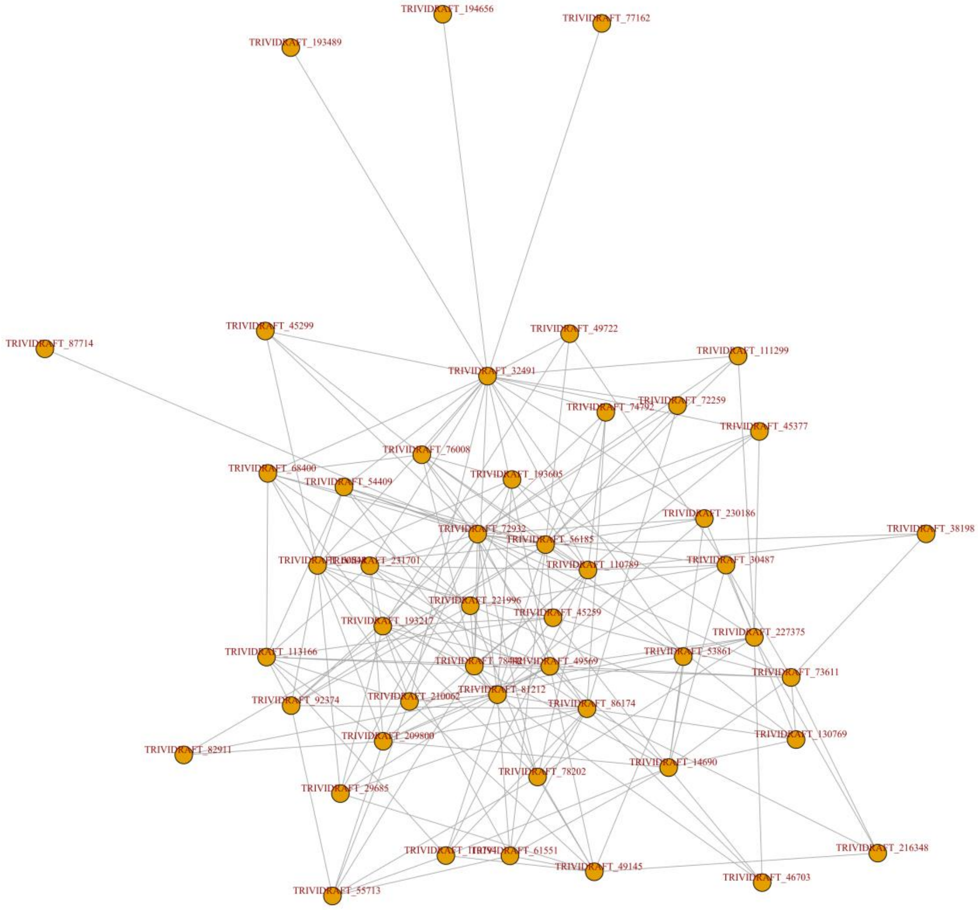

dark turquoise_Hub_network

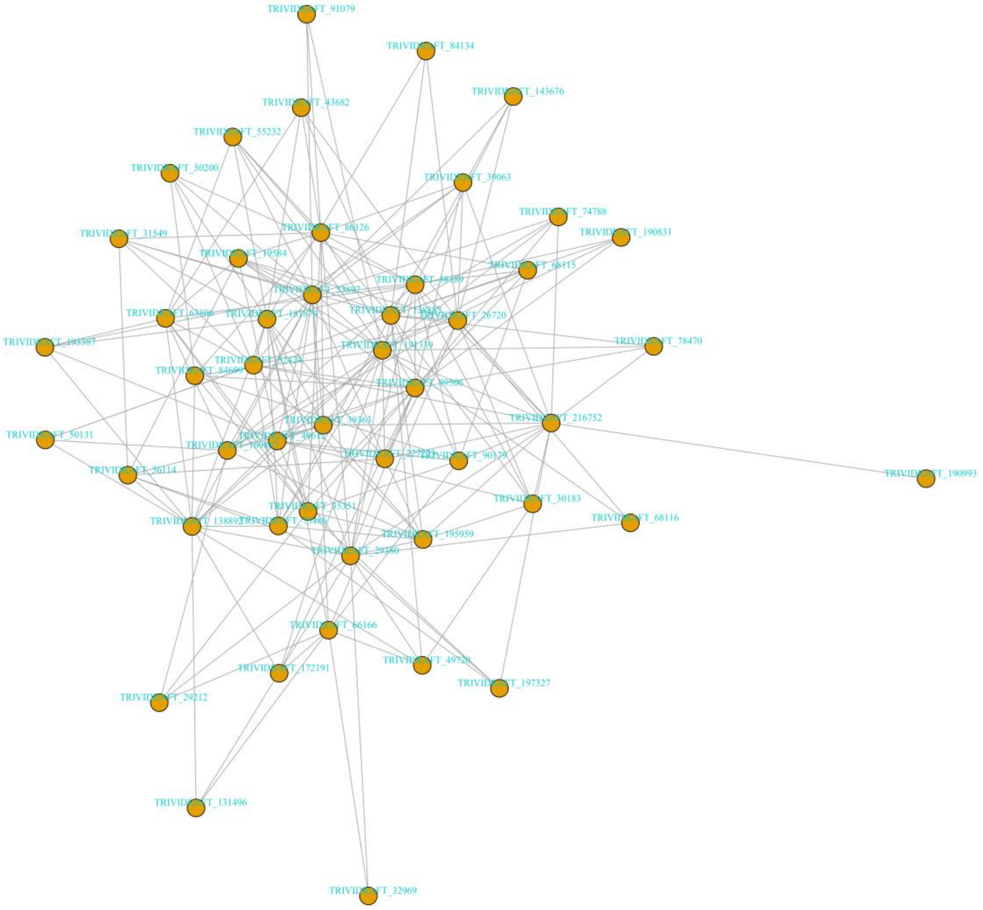

green_Hub_network

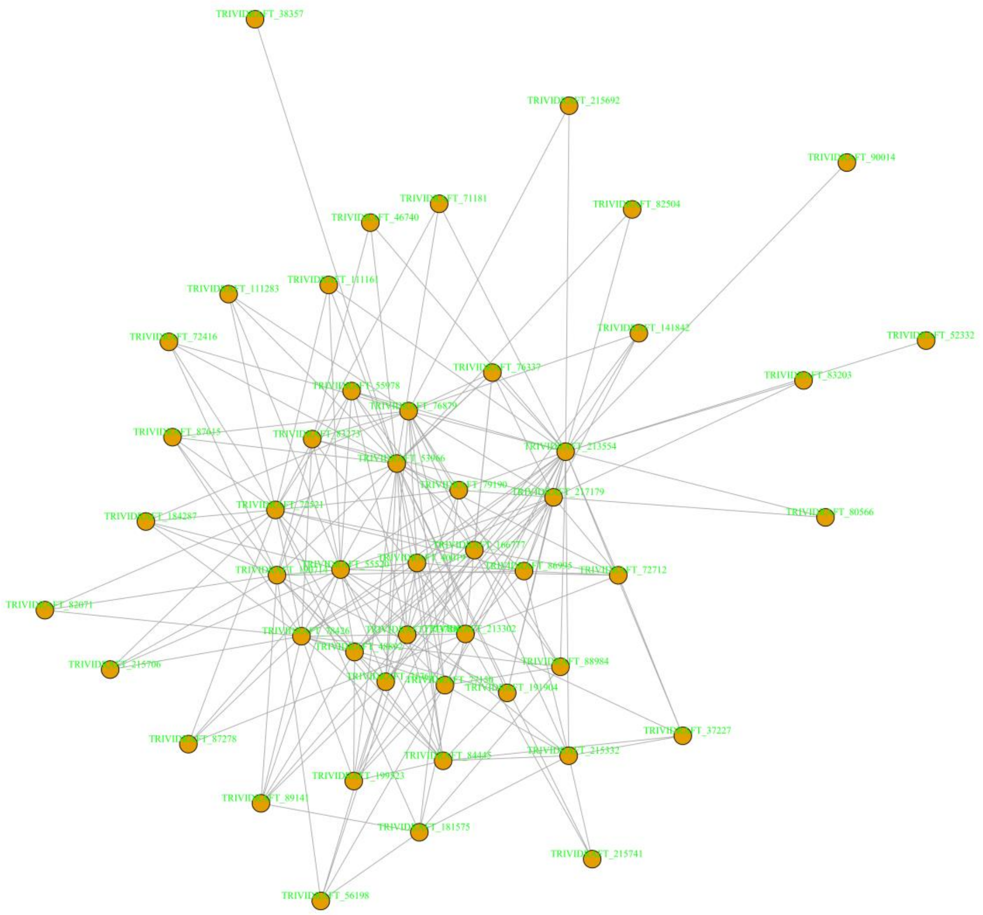

grey60_Hub_network

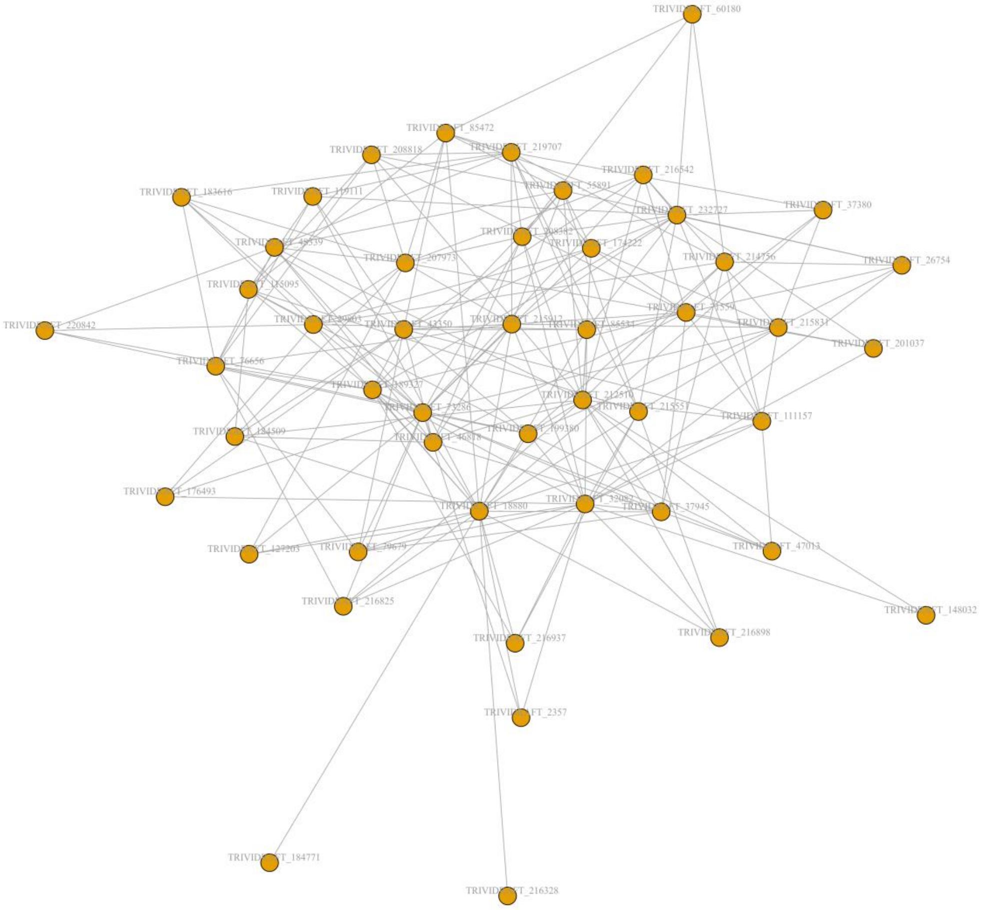

light cyan_Hub_network

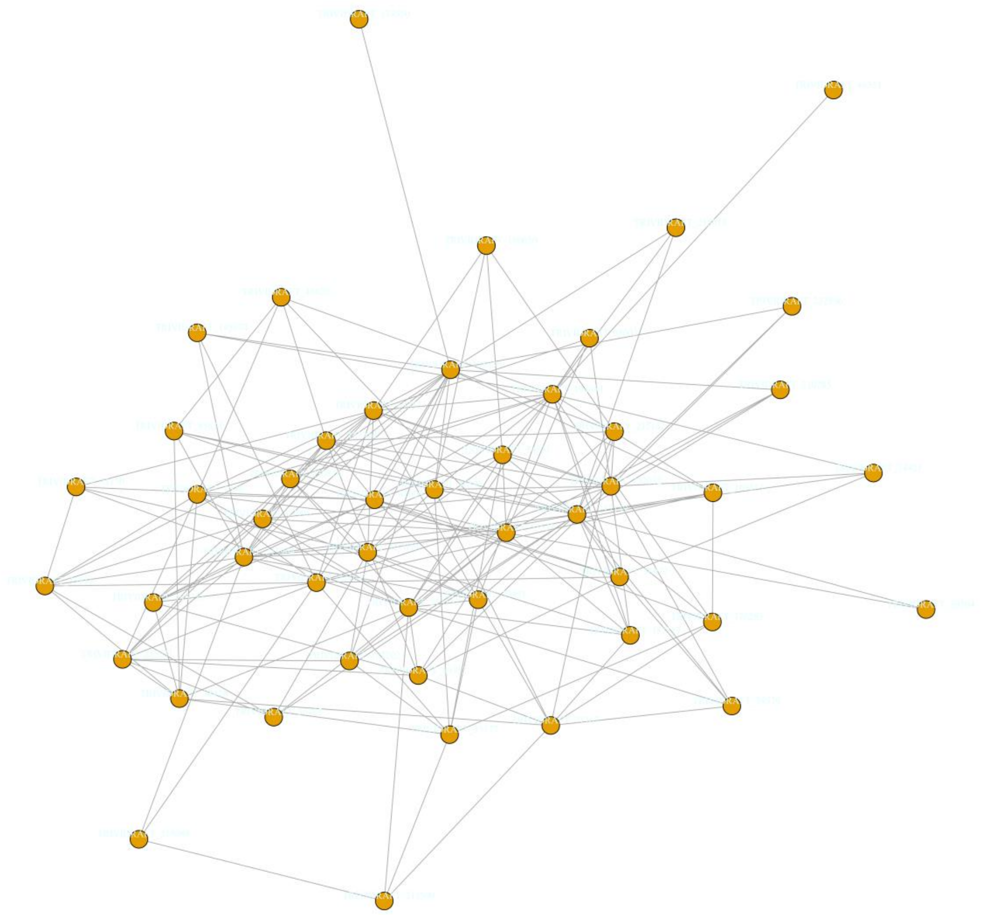

magenta_Hub_network

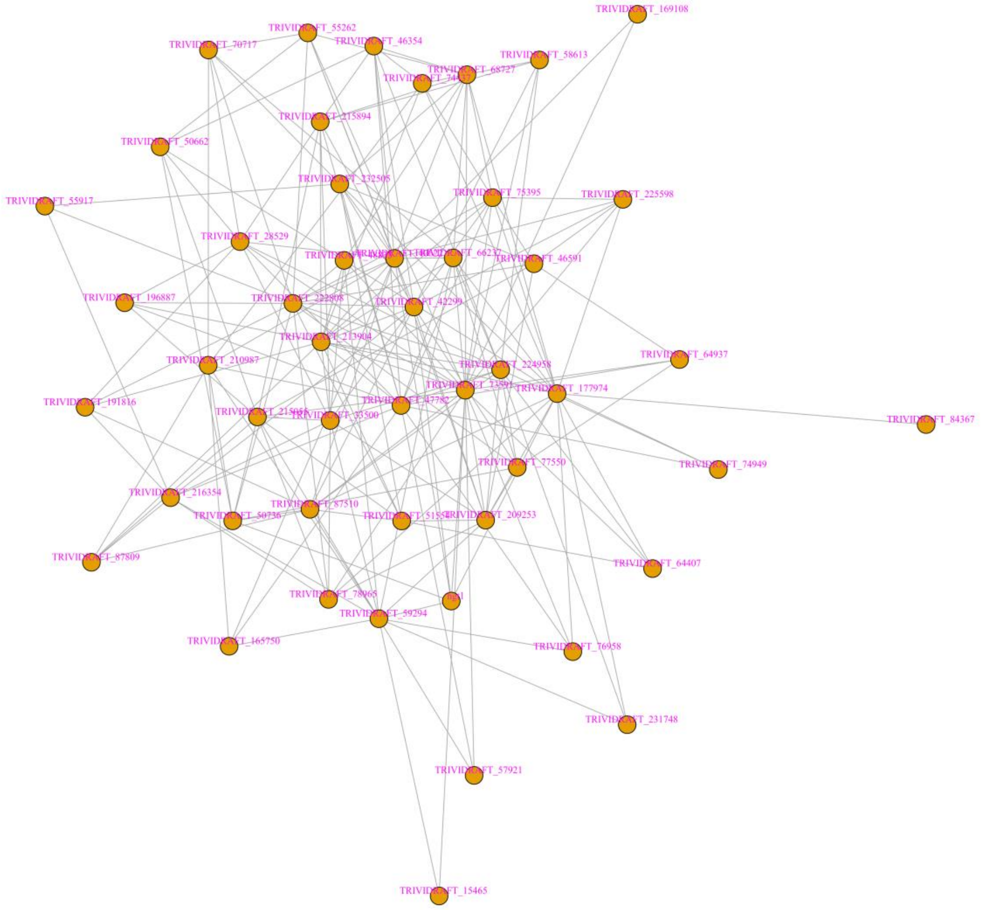

orange_Hub_network

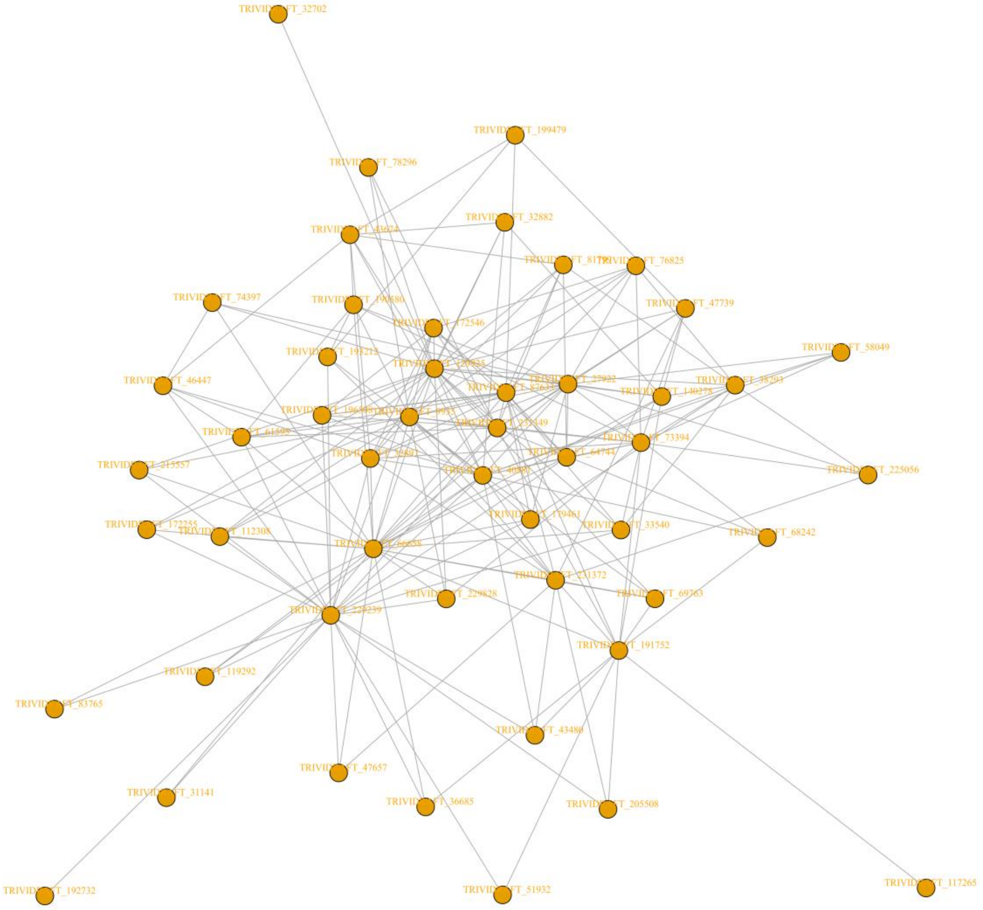

pink_Hub_network

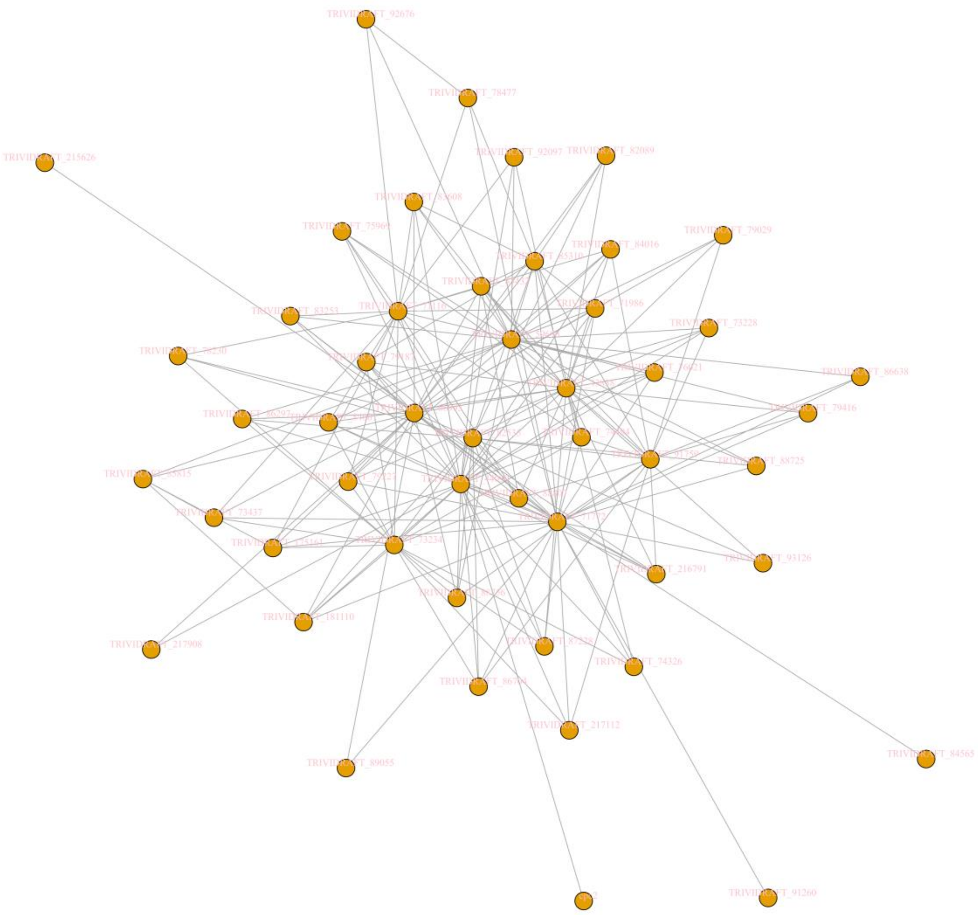

purple_Hub_network

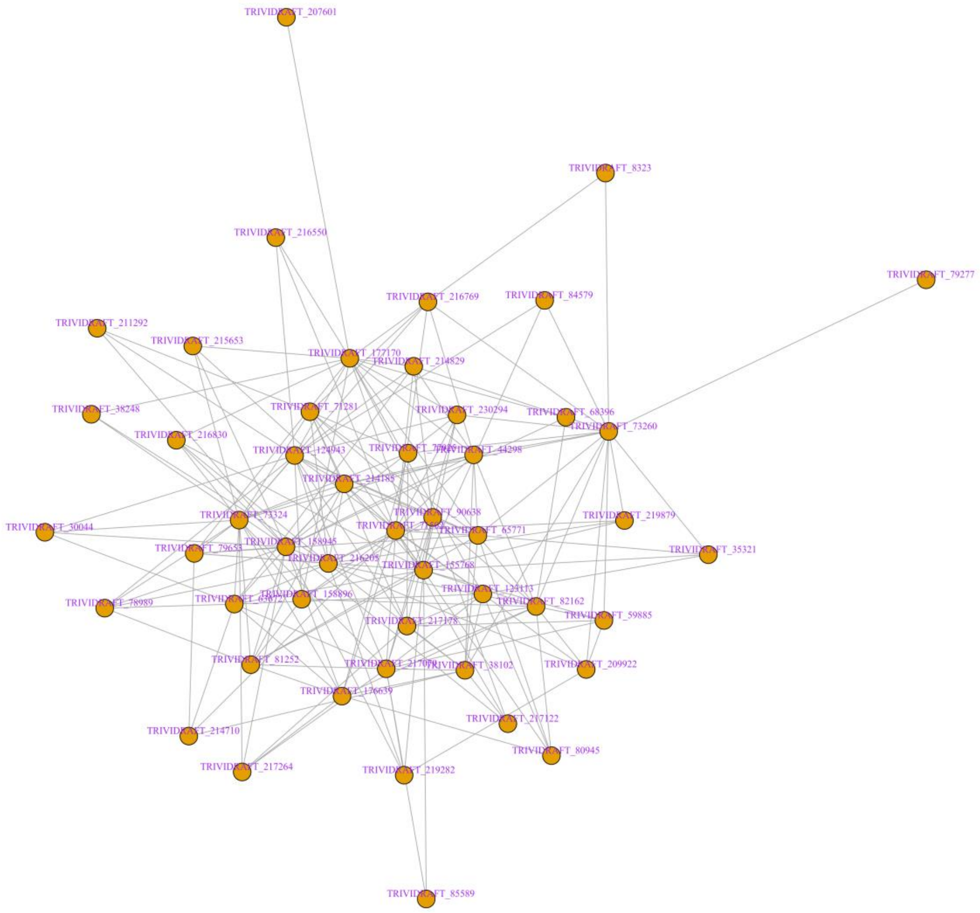

saddle brown_Hub_network

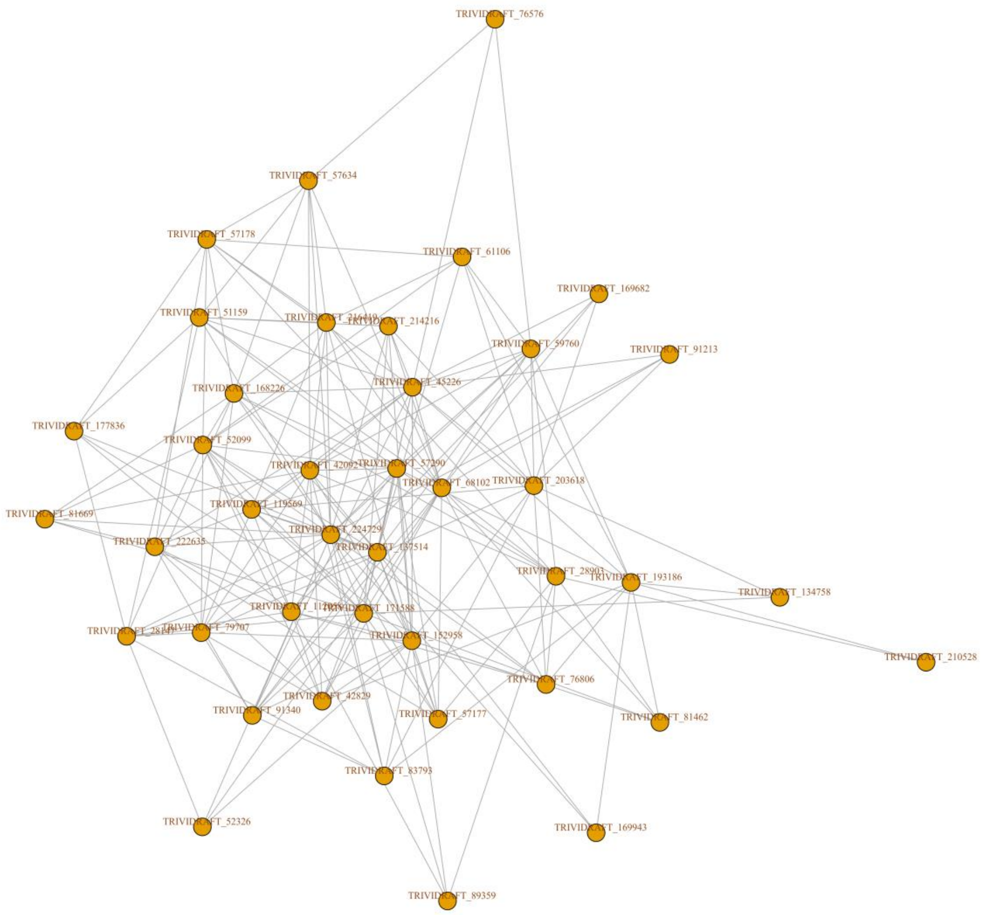

sky blue_Hub_network

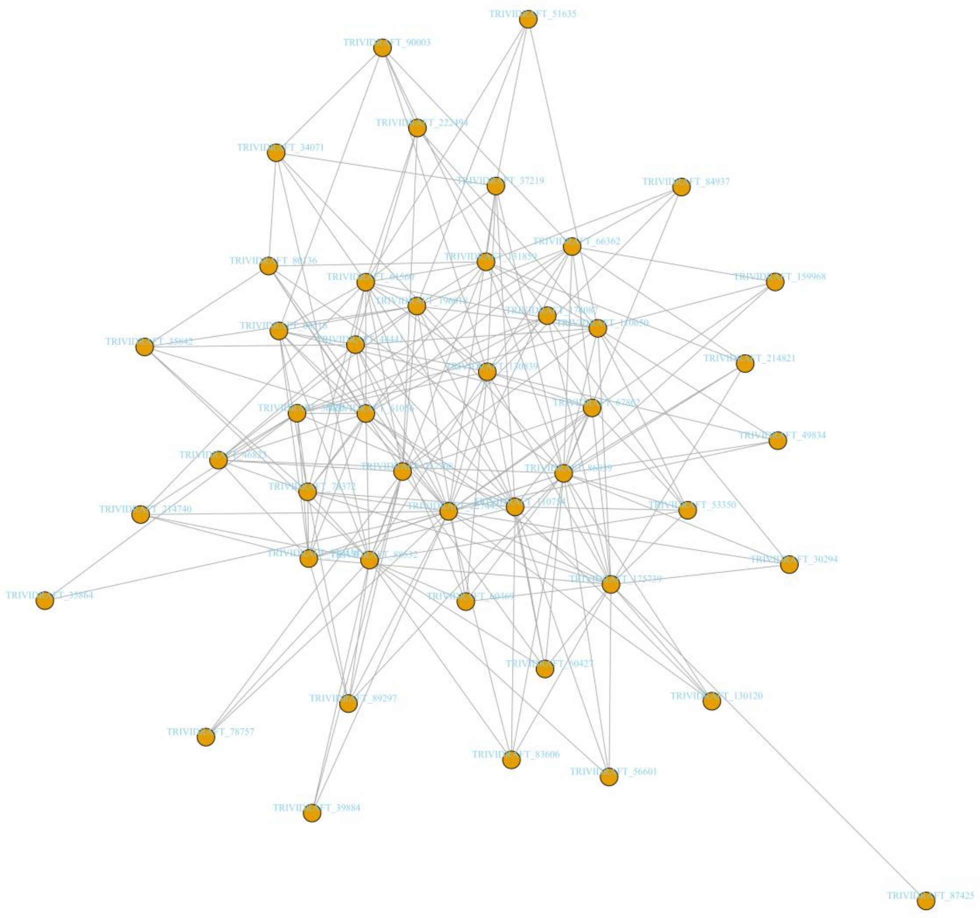

white_Hub_network

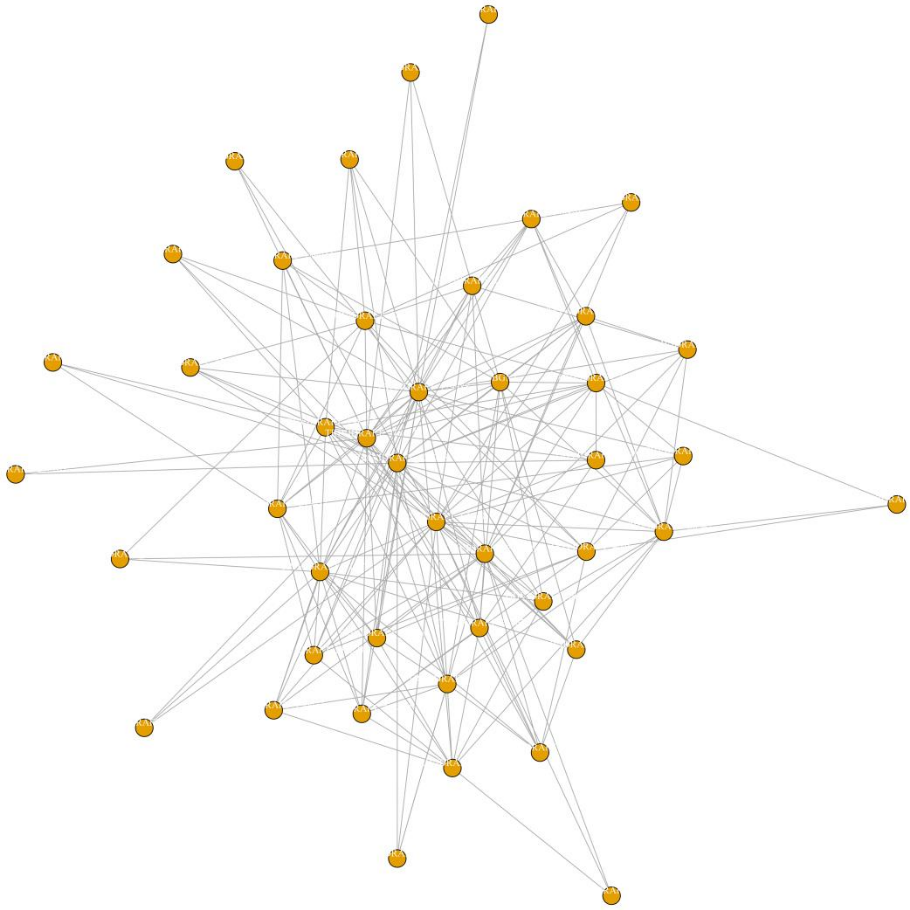

Fig. S6 Protein-protein interaction (PPI) network of genes in the black, dark red, dark turquoise, green, grey60, light cyan, magenta, orange, pink, purple, saddle brown, sky blue and white modules.

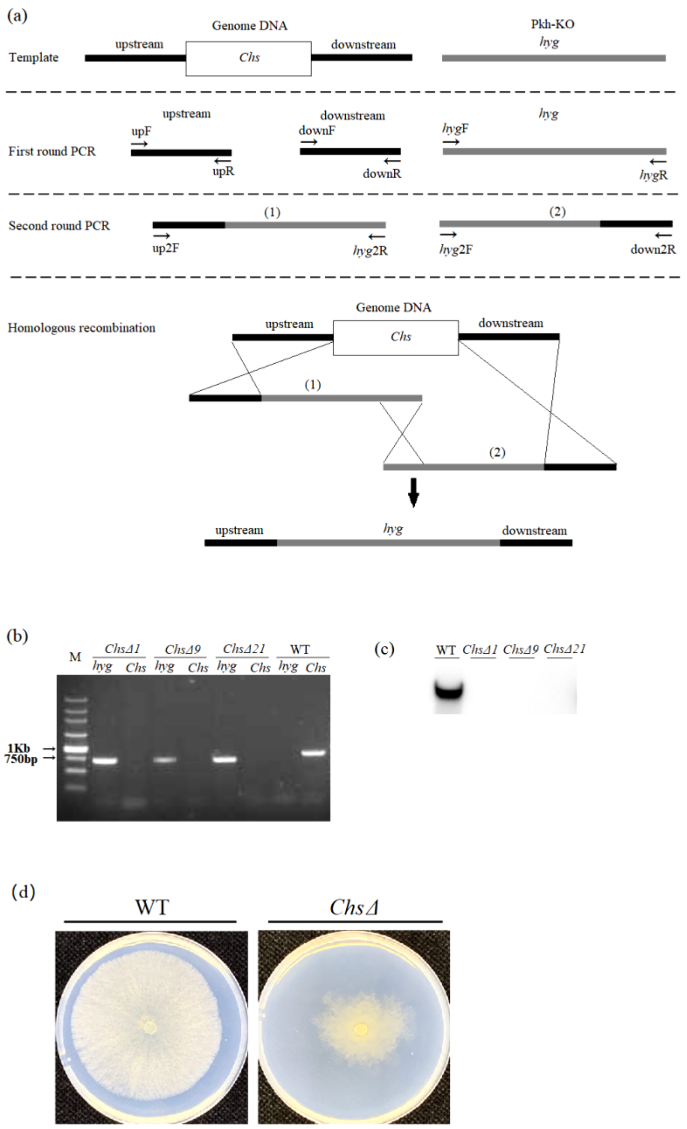

Fig. S7 Construction of chitin synthase gene (TRIVIDRAFT_90152) deletion mutants and the effect on phenotypes of the *T. virens* GV29-8

(a) Chitin synthase gene (TRIVIDRAFT_90152) deleting strategy. The *Chs* gene of *T.virens* GV29-8 was knocked out by split-PCR strategy. The split-marker cassettes were developed using fusion PCR, and involved two rounds of PCR. First round PCR: the upstream and downstream homologous fragments of *Chs* were amplified using primers upF/upR and downF/downR with the genome DNA of *T.virens* GV29-8 as template, respectively. Fragment of the hygromycin gene was amplified with the plasmid pKH-KO as template, and *hyg*F/*hyg*R as primer. Second round PCR: the upstream homologous fragment of *Chs* gene and the *hyg* gene fragment from the first round of PCR products were simultaneously used as templates, and the final fragment (1) was amplified by using the primer up2F/*hyg*2R, which contained the upstream homologous region of the *Chs* gene and two thirds of the hygromycin

gene fragment. Similarly, the downstream homologous region of the *Chs* gene and two thirds of the hygromycin gene fragment was amplified (2). There were 554 bp *hyg* gene overlap region between (1) and (2) in the center of *hyg*. (b) PCR identification of *Chs* and *hyg* genes in *ΔChs1*, *ΔChs9*, *ΔChs21* and WT. M was a 250-bp ladder. PCR templates were genomic DNA of *ΔChs1*, *ΔChs9*, *ΔChs21* and wild-type GV29-8 respectively. (c) Southern hybridization analysis. Probe: *Chs*, DNAs of wild-type GV29-8, *ΔChs1*, *ΔChs9* and *ΔChs21* were digested by BamHI/NotI. (d) Phenotypes of the wild-type *T. virens* GV29-8 and *ChsΔ* mutant strains.

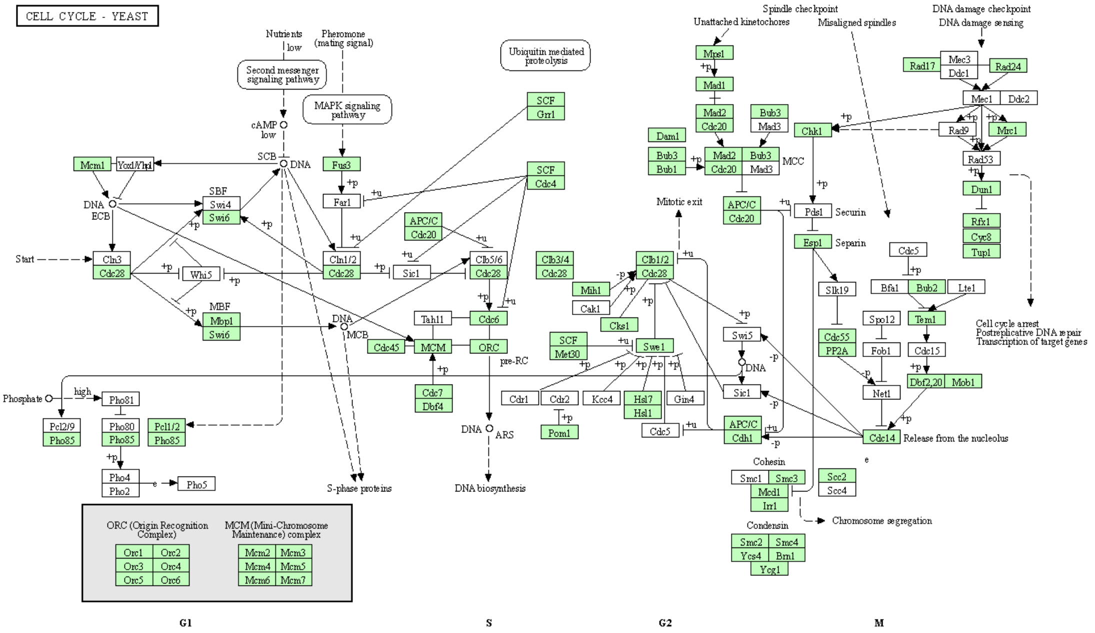

Fig. S8 Cell cycle-yeast pathway (tre04111). DEGs in cell cycle-yeast pathway: Apc/c: anaphase-promoting complex subunit 8 (25794716); CDC45: cell division control protein 45 (25786744); MCM: DNA replication licensing factor MCM3 (25797744/25797702/25797864); CDC7: cell division control protein 7 (25795105); Pom1: dual specificity tyrosine-phosphorylation-regulated kinase (25791399); Met30: F-box and WD-40 domain protein MET30 (25795627); Smc2: structural maintenance of chromosome 2 (25797454); Smc4: structural maintenance of chromosome 4 (25798962); Mcm2: DNA replication licensing factor MCM2 (25797864); Mcm6: DNA replication licensing factor MCM6 (25797744); Mcm7: DNA replication licensing factor MCM7 (25797702); Orc1: origin recognition complex subunit 1 (25793997); Orc6: origin recognition complex subunit 6 (25787414); Mcm4: DNA replication licensing factor MCM4 (25798055). The green box represents a specific gene or enzyme in *Trichoderma Reesei* and does not indicate up-regulation or down-regulation of the gene.

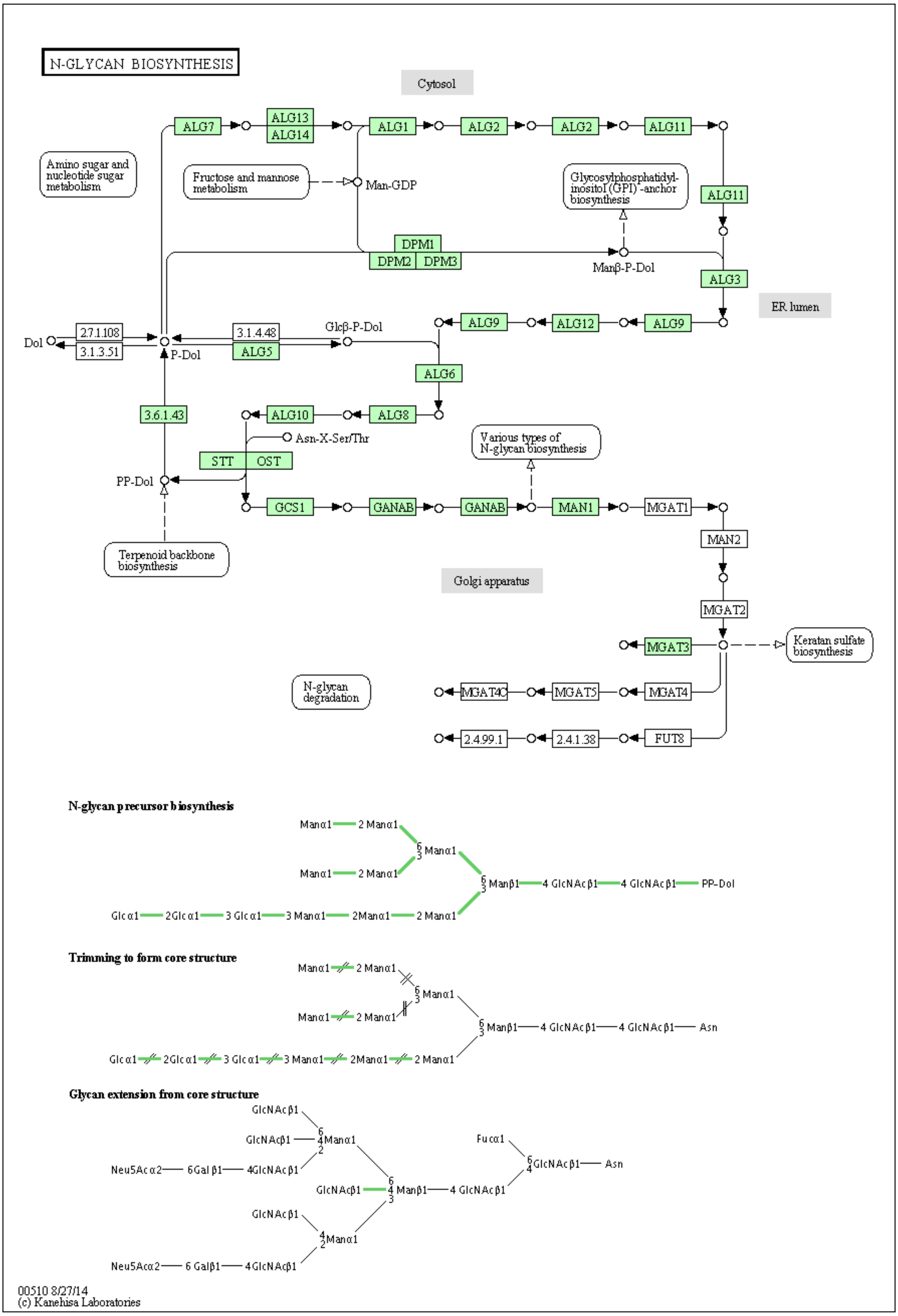

Fig. S9 N-Glycan biosynthesis pathway (tre00510). DEGs in N-Glycan biosynthesis pathway: 3.6.1.43: dolichyldiphosphatase (25792540); DMP1: dolichol-phosphate mannosyltransferase (25798517); ALG5: dolichyl-phosphate beta-glucosyltransferase (25795393); OST: oligosaccharyltransferase complex subunit alpha (25786852/25787193/25798795); MAN1: mannosyl-oligosaccharide alpha-1,2-mannosidase (25798667/

25789754/25795445); STT: dolichyl-diphosphooligosaccharide-protein glycosyltransferase (25797749); ALG1: beta-1,4-mannosyltransferase (25795163); GCS1: mannosyl-oligosaccharide glucosidase (25793447); DPM3: dolichol-phosphate mannosyltransferase subunit 3 (25799030), beta-1,4-N-acetylglucosaminyltransferase (25793326). The green box represents a specific gene or enzyme in *Trichoderma Reesei* and does not indicate up-regulation or down-regulation of the gene.

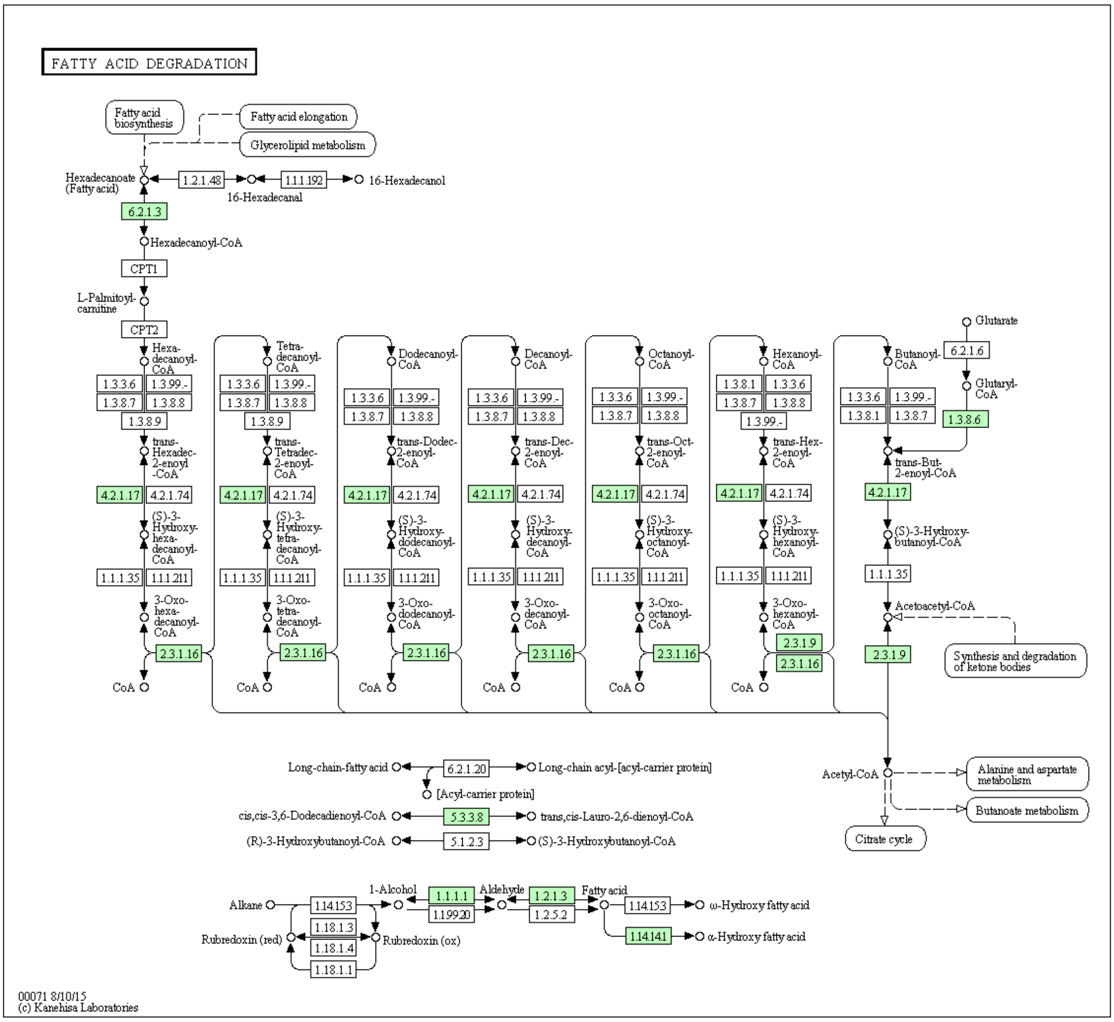

DEGs in fatty acid degradation pathway: 4.2.1.17: enoyl-CoA hydratase (25798760); 2.3.1.9: acetyl-CoA C-acetyltransferase (25788734/25797542); 5.3.3.8: Delta3-Delta2-enoyl-CoA isomerase (25795747); 1.1.1.1: alcohol dehydrogenase, propanol-preferring (25793381); 1.3.8.7: acyl-CoA dehydrogenase (25788983).

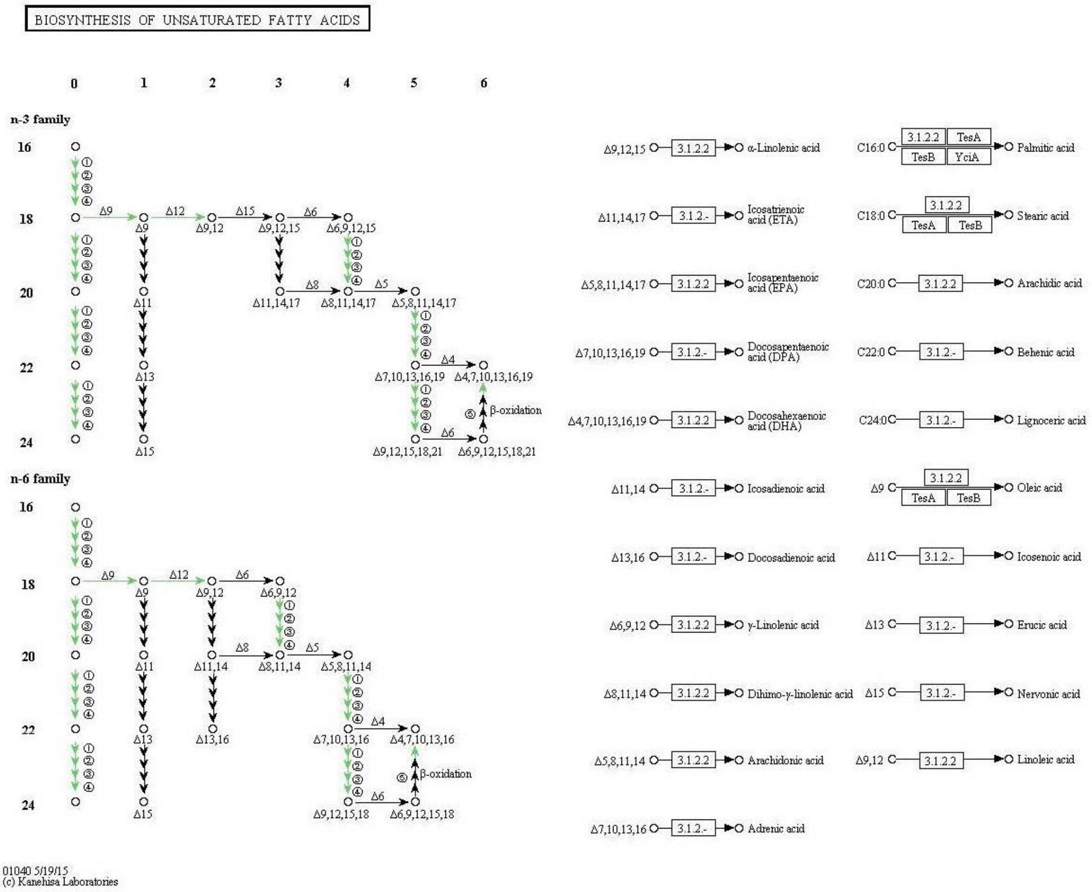

DEGs in biosynthesis of unsaturated fatty acid pathway: ① 2.3.1.199: fatty acid elongase 3 (25798319/ 25794969); ② 1.1.1.62/1.1.1.330: 17 beta-estradiol 17-dehydrogenase/very-long-chain 3-oxoacyl-CoA reductase (25797972/25798045/25792427); ③ 4.2.1.134: very-long-chain (3R)-3-hydroxyacyl-CoA dehydratase (25796723); ④ 1.3.1.93: very-long-chain enoyl-CoA reductase (25792467); ⑤ 2.3.1.16: acetyl-CoA acyltransferase 1 (25798206/25786723).

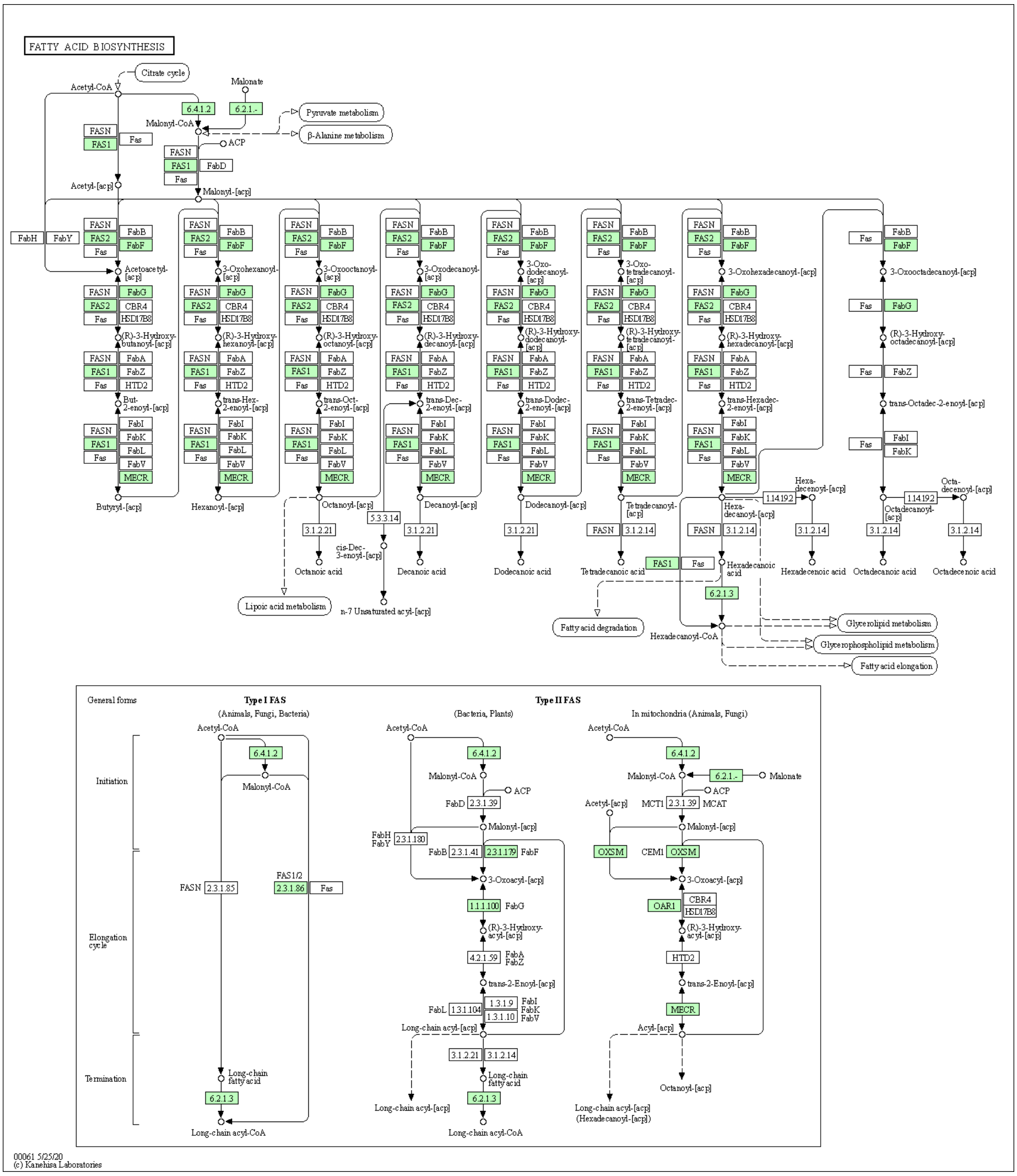

DEGs in fatty acid biosynthesis pathway: 6.4.1.2: acetyl-CoA carboxylase (25798001); 2.3.1.86: fatty acid synthase subunit beta, fungi type (25788772); 2.3.1.179: 3-oxoacyl-[acyl-carrier-protein] synthase II (25798380); 1.1.1.100: 3-oxoacyl-[acyl-carrier protein] reductase (25792427); 6.2.1.3: long-chain acyl-CoA synthetase (25798208).

Fig. S10 Fatty acid metabolic pathway(fatty acid degradation(tre00071), biosynthesis of unsaturated fatty acid (tre01040) and fatty acid biosynthesis pathway (tre00061)). The green box represents a specific gene or enzyme in *Trichoderma Reesei* and does not indicate up-regulation or down-regulation of the gene.

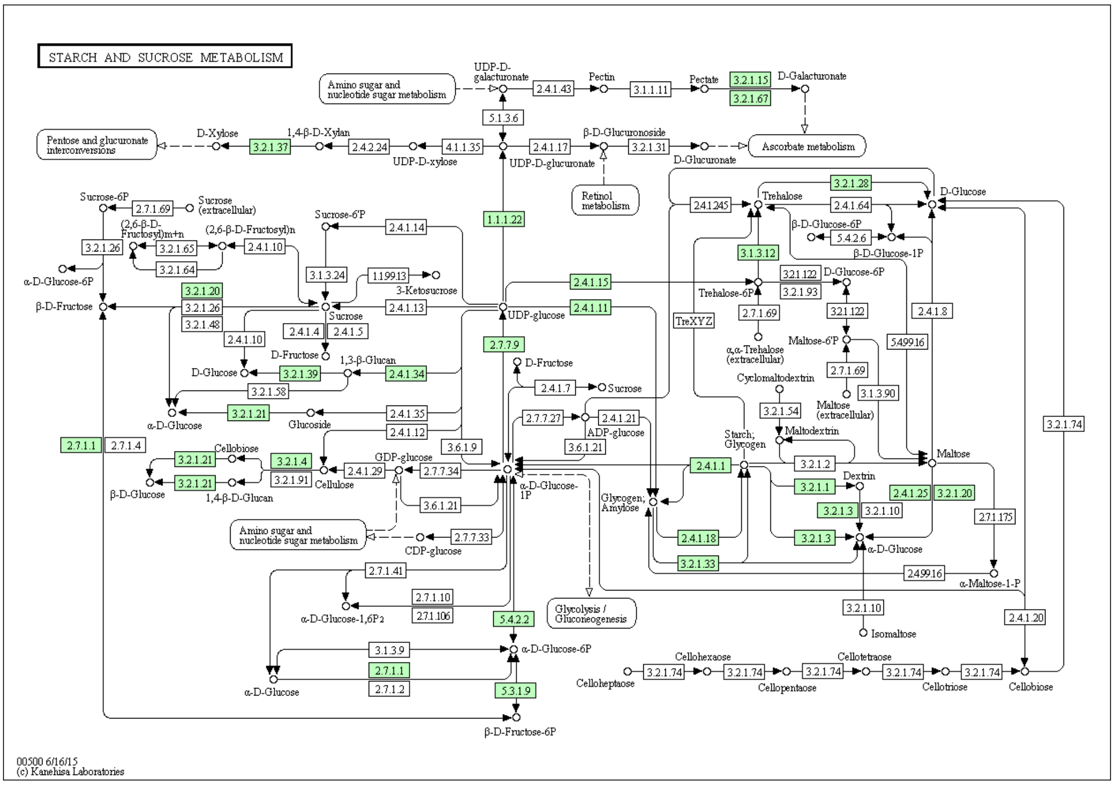

Fig. S11 Starch and sucrose metabolism pathway (tre00500). DEGs in starch and sucrose metabolism pathway: 3.2.1.39: glucan endo-1,3-beta-D-glucosidase (25786810); 3.2.1.21: beta-glucosidase (25797680); 3.2.1.28: alpha,alpha-trehalase (25794082); 5.4.2.2: phosphoglucomutase (25798750). The green box represents a specific gene or enzyme in *Trichoderma Reesei* and does not indicate up-regulation or down-regulation of the gene.

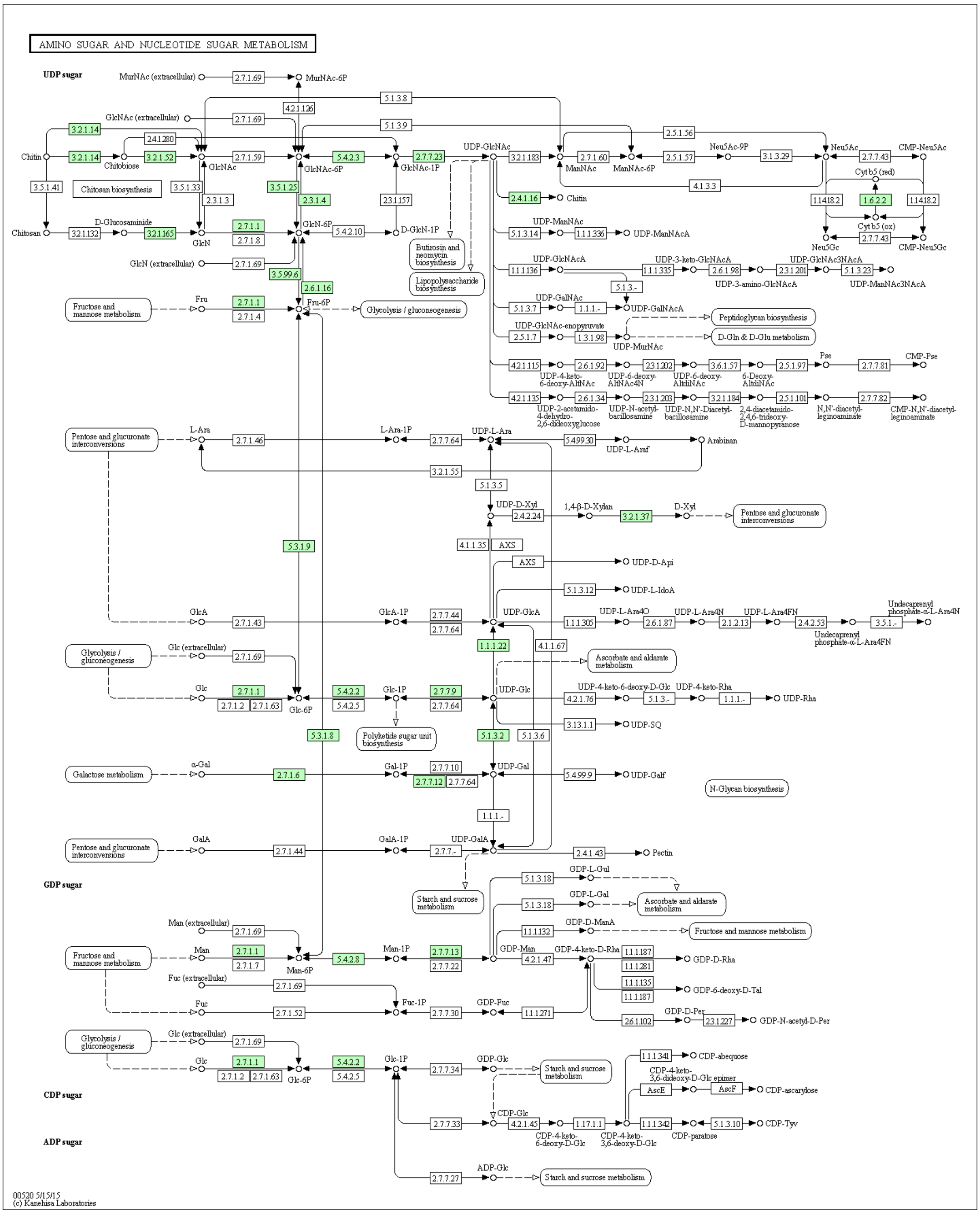

Fig. S12 Amino sugar and nucleotide sugar metabolism (tre00520). DEGs in Amino sugar and nucleotide sugar metabolism pathway: 3.2.1.14: chitinase (25795194/25786774/25786843); 3.2.1.52: beta-N-acetylhexosaminidase (25786802); 5.4.2.3: phosphoacetylglucosamine mutase (25787876); 3.2.1.165: exo-1,4-beta-D-glucosaminidase (25794739); 2.4.1.16: chitin synthase (25798917/25797926/25797171); 2.3.1.4: glucosamine-phosphate N-acetyltransferase (25797997); 5.4.2.2: phosphoglucomutase (25798750). The green box represents a specific gene or enzyme in *Trichoderma Reesei* and does not indicate up-regulation or down-regulation of the gene.
